# Water soluble organic electrochromic materials

**DOI:** 10.1039/d0ra10346b

**Published:** 2021-01-27

**Authors:** Thomas A. Welsh, Emily R. Draper

**Affiliations:** School of Chemistry, University of Glasgow Glasgow G12 8QQ UK emily.draper@glasgow.ac.uk

## Abstract

Organic materials in electrochromic device applications possess a number of advantages over transition metal oxides like WO_3_ such as ease of synthesis and tunability, flexibility, and derivability from renewable feedstocks. However, these advantages are offset by the need to use organic solvents in their processing which are often flammable and/or toxic. Therefore, it is of paramount importance to the longterm economic and environmental sustainability of organic electronics research to develop water soluble organic materials. Herein, we describe the advances made in developing water soluble organic electronic materials for electrochromic applications. We here classify electrochromic materials into two broad categories: those that transition between colourless and coloured states (Type I) and those that transition between differently coloured states (Type II). The methods by which organic electrochromes are made water soluble are described in detail along with their potential applications in order to promote research in water soluble organic electronic materials in general.

## Abstract 2

Here we discuss water soluble organic chromic materials classed as two different types, Type I - colourless to coloured and Type II - coloured to coloured states. This change in colour is vital in their applications.

## Graphical abstract

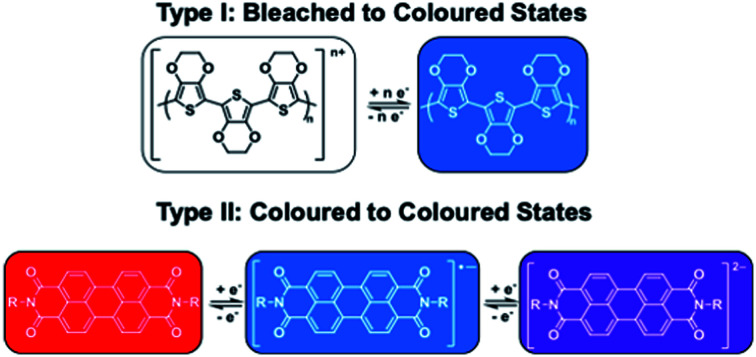

## 1. Introduction

Research in organic electronic materials has increased considerably in the past few decades with applications in photovoltaics,^1–4^ field-effect transistors,^5–7^ light-emitting diodes,^8–10^ sensors,^11,12^ and batteries.^13^ Organic materials possess a number of attractive qualities that make them well suited for electronic device application. Organics can be derived from abundant and/or renewable feedstocks; are lightweight, flexible, and solution processable; and possess physical and optoelectronic properties that can be easily modified *via* simple chemical reactions. One such optoelectronic feature of particular interest is electrochromism, or the ability to change colour upon a change in oxidation state.^14,15^ The types of colour changes an electrochrome undergoes dictate its application. For example, an electrochromic material that changes between a colourless transparent state and a deeply coloured opaque state would be suited for application in smart windows to promote energy efficiency and privacy while an electrochrome that changes between multiple differently coloured states is better applied in sensors and display devices.^16–18^

Another important factor dictating material application is solubility. As mentioned above, solution processability is a key feature of organic materials enabling for ease of device manufacture through large-scale printing.^19^ However, many organic materials are only processable from environmentally unfriendly and often hazardous solvents such as dichloromethane and chloroform.^20^ Non-halogenated organic solvents such as hexanes, toluene, or tetrahydrofuran also pose risks due to their flammability or toxicity.^20^ In order to truly be environmentally sustainable, the ideal processing solvent is water as it is both nontoxic and the most abundant solvent on earth. Unfortunately, many organic compounds have little to no solubility in water. In contrast, inorganic materials are often readily water soluble. There are many electrochromic devices based off metal oxides including W, Cu, Ag, Bi, Pb, among others.^21^ Indeed, WO_*x*_ is one of the most ubiquitous metal oxides used in electrochromic devices but it, along with all other metals, requires mining to source the raw materials, which is very environmentally harmful and unsustainable long term. Organic materials on the other hand can be produced from renewable feedstocks and their toxicity is of much lower concern than many metals like Pb, for example. To fully replace metal oxides with organics simply requires developing water-soluble organic materials.

To make an organic molecule water soluble is not particularly difficult and often merely requires incorporating a charged functional group such as carboxylates, sulfonates, or ammoniums. Other materials may be inherently water soluble by being charged or becoming charged through redox processes. Materials that are not water soluble can also be combined with other materials that are water soluble to make water soluble dispersions. The advantages to developing water soluble organic electrochromic materials are the same as developing water soluble organic electronic materials in general. For electrochromes that have application in smart windows or display systems, water solubility is particularly important for reducing hazardous waste and manufacturing costs. When focusing on electro-active materials, the addition of such functional groups can have huge consequences on the electronic properties of the materials, as well as the aggregation of the materials. This change in solubility in turn changes more than just the hydrophilicity of materials, but also the absorption properties and reduction/oxidation potentials. Therefore, it is important to have a comprehensive understanding of the effects of water solubility on optoelectronic properties in organic electro-active materials. In our research we have noticed that, while there are many reviews covering electrochromic materials and water soluble materials, there are none that cover materials that are both electrochromic and water soluble. Herein we aim to provide such a review by discussing electrochromic water soluble polymers and molecules. We will begin by providing a brief description of the properties by which electrochromic materials are characterized and then discuss various types of electrochromic materials. It is our hope to shine a light on an area that has important implications for improving sustainability in materials research.

## 2. Electrochromism

**Fig. 1:**
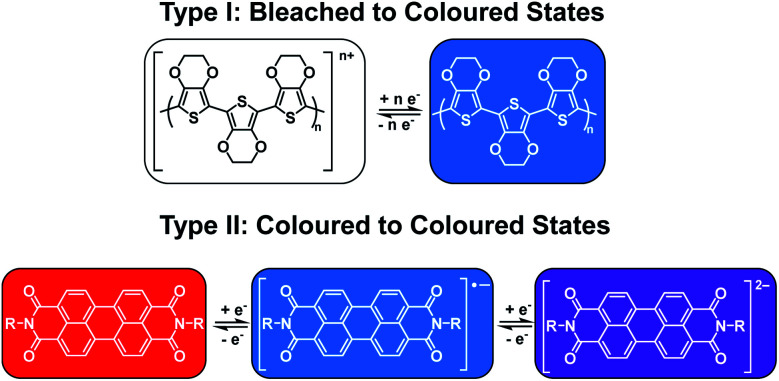
The two types of electrochromic materials discussed in this review.

As mentioned above, electrochromism in organic compounds is a change in colour upon a change in oxidation state. In other words, an electrochromic material is a conductive material that changes colour when a specific current or potential is applied. These materials can be grouped into two broad categories (Fig. 1). The first category (Type I) involves materials that possess at least one coloured state and at least one colourless or ‘bleached’ state.^22^ Examples include polymers such as poly(3,4-ethylenedioxythiophene) (PEDOT) and poly(3,4-propylene-dioxythiophene) (PProDOT) and small molecules such as viologens.^14^ The materials' neutral state can be coloured or bleached and the switch can be achieved by either oxidation or reduction. Such materials are typically applied in smart windows where a change from colourless to coloured is desirable.^15^ The second category (Type II) involves materials that lack a bleached state and possess two or more differently coloured states. Examples include polythiophenes (PTs) which change from red to blue upon oxidation as well as perylene diimides (PBIs) which are red, blue, and violet in the neutral, radical anion, and dianion states, respectively.^14,22,23^ These types of materials find more use for display purposes.^15^Table 1 lists the Type I and Type II electrochromes that will be discussed in this review.

**Table 1:** The electrochromic materials discussed in this review along with the potentials used to achieve the oxidized and reduced states and the method by which the materials are made water soluble. The colours as described by the authors are provided in brackets

Material	Oxidation potential (V)	Reduction potential (V)	Method of water solubility	Ref.
**Type I**				
PEDOT/PSS	2.0 (sky blue/colourless)	−2.0 (blue)	Soluble dispersion	24 and 25
PProDOT-propargyl	1.0 (colourless)	−1.0 (dark blue)	Soluble functional groups	26
PProDOT-acid	0.55 (colourless)	−0.50 (purple)	Functional group deprotonation	27
PProDOT-BT	0.5 (colourless)	0.0 (dark blue)	Functional group deprotonation	28
PPE	0.7 (colourless)	0.0 (blue)	Functional group deprotonation	29
Regiosymmetric PProDOT	0.5 (colourless)	0 (blue)	Functional group deprotonation	30
MV^2+^/GQD	0.0 (colourless)	−2.8 (purple)	Charged material	31
EV^2+^/PVA-borax slime	0.0 (colourless)	−2.3 (violet)	Charged material	32
CPV^2+^/PVA-borax slime	0.0 (colourless)	−0.5 (green)	Charged material	33
		−0.9 (purple)		
BVA^2+^	0.0 (pale yellow)	−0.3 (dark violet)	Charged material	34
DHPV^2+^/PVB gel	0.0 (colourless)	−2.0 (dark blue)	Charged material	35
DHPV^2+^/PVA gel	0.0 (colourless)	−2.0 (dark violet)	Charged material	35
SEV	0.0 (pale yellow)	−2.2 (rose red)	Charged functional group	36
SPV	0.0 (pale yellow)	−2.2 (purple)	Charged functional group	36
NDI-GF	0.6 (colourless)	−0.7 (black)	Functional group deprotonation	37

**Type II**				
PT34bT/PSS	— (green)	— (blue)	Soluble dispersion	38
PEDOT-S/PAH	0.6 (light blue)	−0.8 (pink purple)	Charged functional group	39
PEDOT-CH_2_NH_3_^+^A^−^	0.8 (blue)	−0.5 (red)	Charged functional group	40
PANI	— (violet)	−0.2 (pale yellow)	Charged material	41 and 42
	0.6 (blue/green)			
PANI-SWCNT	2.0 (sky blue)	−2.0 (green yellow)	Charged material	43
PANI-Au	0.6 (sky blue)	0.0 (green yellow)	Charged material	44
PBI-A	0.0 (red)	−1.0 (purple)	Functional group deprotonation	45
		−2.5 (navy blue)		
PPBI	0.0 (orange red)	−1.3 (purple)	Charged functional group	46
Phenol red hydrogel	0.0 (yellow)	−0.5 (purple)	Charged material	47
Thymol blue hydrogel	0.0 (yellow)	−0.5 (blue)	Charged material	47

In this section we will briefly discuss some common parameters used to characterize electrochromic materials and electrochromic devices (ECDs). A more detailed description of these parameters can be found in the literature.^21,22^

### 2.1. Optical contrast and colouration efficiency

As electrochromism is defined as change in colour between oxidation states, a precise method of determining a change in colour is required. This is known as electrochromic or optical contrast (OC) or percent transmittance change (Δ*T*). It is the primary tool by which electrochromic materials are characterized and involves monitoring at one or more wavelengths the change in transmittance during oxidation or reduction.^21,22^ Measurements typically involve applying square-wave potential steps and recording the transmittance values at wavelengths that represent the highest contrast between the two oxidation states. The difference in transmittance between the two states will provide the optical contrast given as a percent. Optical contrast is typically a voltage-dependent parameter as higher applied voltages can lead to greater differences in colour.

In addition to optical contrast, electrochromic materials are also characterized by colouration efficiency (CE). Measured in cm^2^ C^−1^, CE is a proportionality factor between the observed change in absorbance (Δ*A*) and the charge density necessary to induce the change (*Q*_d_, C cm^−2^). As materials differ in how intensely coloured they are at their various oxidation states, CE is dependent on a material's inherent properties as well as being voltage dependent. Quantitatively, CE is determined by the equation derived from the Beer–Lambert Law:^22^1
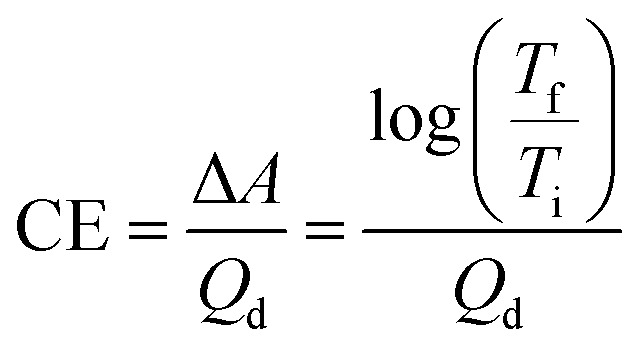
where *T*_f_ and *T*_i_ are the transmittances of the final and initial oxidation states, respectively. As eqn (1) relates the transmittance values at two differing oxidation states, CE is most useful for characterizing Type I materials where the transmittance change between coloured and bleached states is most pronounced. For Type II electrochromic materials that switch between two differently coloured states, CE is less useful for characterization and a low CE value does not necessarily reflect a subtle change in colour.

### 2.2. Switching rates and stability

Another important characterization parameter for electrochromic materials, particularly as it relates to device performance, is switching rate, which is the time it takes for a material to switch from one redox state to another.^21,22^ The time for the redox event is usually measured at 90–95% of transmittance change in academic literature as the typical human eye cannot perceive a difference in the final 5–10% of transmittance change. In industry the standard is 85% of transmittance change. For Type I electrochromic materials the time for the material to go from the bleached state to coloured state is known as the colouration time (*t*_c_) while the reverse is the bleaching time (*t*_b_) while for Type II materials just switching time (*t*) is referenced. Switching times are often dependant on device architecture as much as they are on the inherent optoelectronic properties of the materials and can be influenced by electrolyte, film thickness and morphology, electrochemical cell configuration, and extent of external bias.^22^

Related to switching time is the material stability, which is important for widespread and long-term material application. Stability is typically determined by constantly switching between redox states on the order of 10^3^ to 10^6^ cycles *via* repeated potential cycling or applying potential steps and measuring how the electrochromic contrast changes over time. During a stability measurement the time for the applied potential is typically set a few seconds longer than the switching time in order for the full electrochromic contrast to occur. An irreversible redox event under high potentials will lead to degradation and subsequent loss of electrochromic contrast. Similarly, a partially irreversible redox event, while not resulting in material degradation, will also lead to a gradual loss in electrochromic contrast. Using a sealed or encapsulated device can improve material stability by preventing solvent evaporation and air-induced oxidation reactions. Stability is also influenced by issues with device architecture as much as it is by material properties. Thus, low cyclability and loss of optical contrast may be due to mechanical problems rather than with the materials themselves.

### 2.3. Colourimetry

**Fig. 2:**
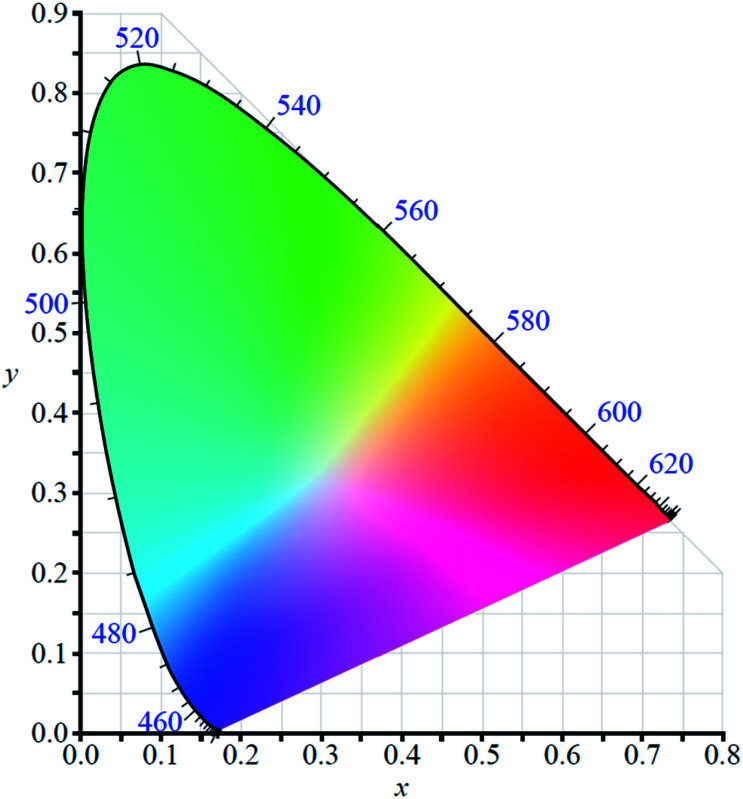
The CIE 1931 colour space chromaticity diagram.

In order to use any coloured compound in a multichromic display application it is necessary to numerically define its colour value.^48^ For electrochromic materials Reynolds *et al.* have established a method by which to describe the different coloured states based on the Commission Internationale de l'Eclairage (CIE) standards.^49,50^ Colour is described quantitatively using three parameters which depend on film thickness and light source: L*, a*, and b*. The L* is brightness while a* and b* are axes representing green (−a*) to red (+a*) and blue (−b*) to yellow (+b*). By using the CIE's colour space coordinate system (Fig. 2), the precise colour value of the material at different oxidation states can be defined and the path the material charts through the coordinate system as it switches between states can also be mapped, thereby providing insight into the material's optoelectronic properties. This method has proven useful in characterizing Type II electrochromic materials and in understanding the colour changes occurring in multilayer ECDs.

## 3. Type I: bleached to coloured materials

As mentioned above, organic electrochromic materials can be classified into two types: those that change between a colourless or bleached state and a coloured state (Type I) and those that change between two or more differently coloured states (Type II). This section will discuss examples of Type I electrochromic materials. Type I materials find use in smart window technology where a change from colourless and transparent to coloured and transparent or opaque is desirable to control lighting and energy needs or privacy concerns. Other applications include in sensor technology. Most Type I materials are colourless in the neutral or oxidized state and coloured in the reduced state. Because they are defined by a change between colourless, low-absorbing states and coloured, highly absorbing states Type I materials are most often characterized by optical contrast, colouration efficiency, and colouration and bleaching times. These properties can be easily defined when large contrasts in absorption between different oxidation states are present, as is the case for Type I materials. Type I materials come as both polymers and molecules, the most common being polythiophene derivatives and viologens.

### 3.1. Type I polythiophene derivatives

**Fig. 3:**
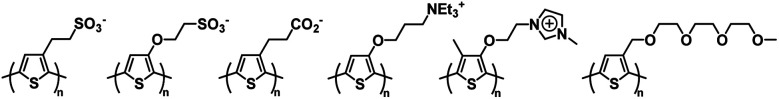
Water soluble polythiophenes with anionic, cationic, and neutral solubilizing functional groups.^53,54^

Electrochromic polymeric materials are well known in the literature.^22,51^ As mentioned above, one of the most ubiquitous types of conjugated polymers, polythiophene and its derivatives, are electrochromic. There are also many examples of water soluble polythiophene derivatives and their applications in chemical and biological sensors, photovoltaics, and field effect transistors are well documented.^52–54^ Polythiophene is usually made water soluble by incorporating charged functional groups. These can be anionic such as sulfonates and carboxylates or cationic such as ammoniums or imidazoliums.^53^ Hydrophilic neutral functional groups such as glycol can also be incorporated to make the polymers water soluble.^54^ Some examples of water soluble polythiophenes can be found in Fig. 3. Polythiophene itself is a Type II electrochromic material and displays red to blue electrochromism upon oxidation.^22,55^ Many derivatives of polythiophene, such as PEDOT and PProDOT are Type I and examples of these will be outlined in this section.

**Fig. 4:**
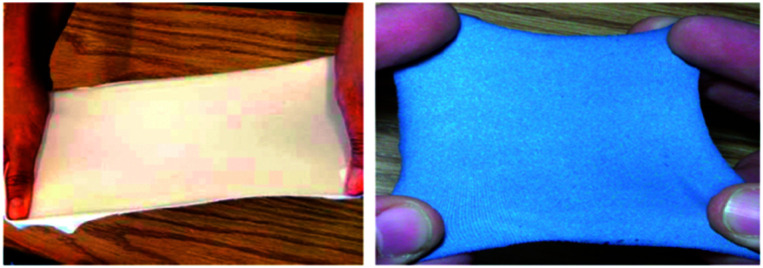
Stretchable Spandex fabric before (left) and after (right) soaking in PEDOT/PSS. Adapted with permission from Y. Ding, M. A. Invernale and G. A. Sotzing, *ACS Appl. Mater. Interfaces*, 2010, **2**, 1588–1593. Copyright 2010 American Chemical Society.

PEDOT is a commonly used polymer in organic electronic research owing to its ease of synthesis and functionalization and favourable optoelectronic properties, such as turning blue from colourless upon reduction.^56^ It is insoluble in water but is often paired with the polyelectrolyte polystyrene sulfonic acid (PSSA) to form a PEDOT/PSS dispersion in water. As a dispersion PEDOT exits in the cationic oxidized state and is colourless to pale blue in colour.^56^ When reduced to the neutral state it is a deep blue. The ease with which PEDOT can be made water processable and its inherent electrochromism has made it a common polymer to use when developing and testing new types of electrochromic technologies and devices. For example, an early example of a functional electrochromic material comes from the Sotzing group who in 2010 reported on a method to make electrochromic fabrics using commercially available PEDOT/PSS.^24^ They found that soaking a variety of fabrics for 5 minutes in a PEDOT/PSS aqueous dispersion doped with 2 wt% d-sorbitol led to light blue fabric that could be reversibly bleached upon oxidation (Fig. 4). The fabric also retained its original flexibility and stretchiness upon PEDOT adsorption. Stretching the fabrics led to a decrease in conductivity but electrochromic behaviour was otherwise maintained, even while stretched. Overall, this study demonstrated the potential to create wearable electrochromic fabrics, an important step in demonstrating new applications for these types of materials.

**Fig. 5:**
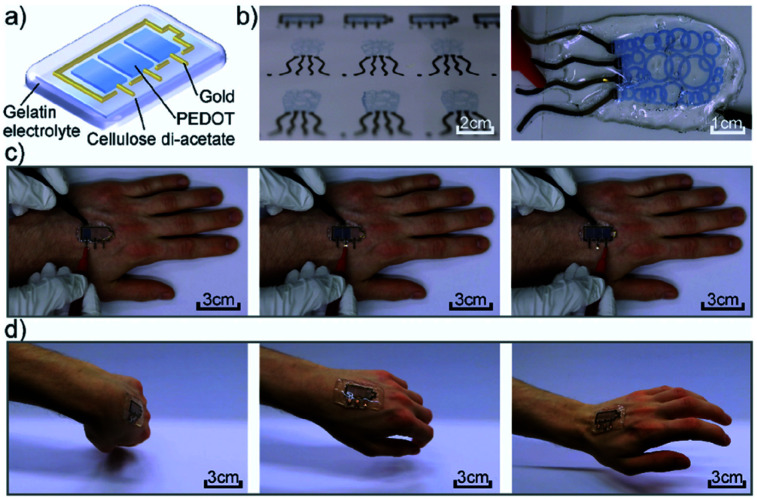
(a) Device architecture, (b) printed devices, (c) operation of the device while attached to the hand, and (d) demonstration of device flexibility while moving the hand. M. Pietsch, S. Schlisske, M. Held, N. Strobel, A. Wieczorek and G. Hernandez-Sosa, *J. Mater. Chem. C*, 2020, Advance Article, DOI: 10.1039/D0TC04627B – Published by The Royal Society of Chemistry.

Another more recent example of a functional electrochromic device that incorporates water soluble PEDOT/PSS dispersions comes from the Hernandez-Sosa group who have reported on an inkjet printed biodegradable wearable electrochromic device.^25^ The flexible and device architecture they developed involves PEDOT/PSS pixels connected to gold working electrodes and surrounded by a gold counter electrode, all of which is contained in a biocompatible gel electrolyte based on natural salts and gelatin-based hydrogel (Fig. 5). A variety of natural salts including NaCl, CaCl_2_, and K_2_SO_4_ were tested and they found that NaCl provided the highest contrast at low voltages with an OC of 32 ± 4% at 685 nm, a CE of 99 ± 6 cm^2^ C^−1^, and switching times of 3.0 ± 1.4 s for printed devices. These figures are comparable with a reference device prepared *via* spin coating. As the devices are intended to be wearable, as shown in Fig. 5, they were also subjected to stretch testing and it was found that after 10 000 bending cycles the devices only lost about 15.9% of OC from the initial value. Overall, this study demonstrated the ease with which a flexible electrochromic device could be made with environmentally friendly and biodegradable water soluble components. Furthermore, the ability for the devices to be manufactured *via* inkjet printing allows for ease of scale-up production while minimizing waste.

**Scheme 1:**
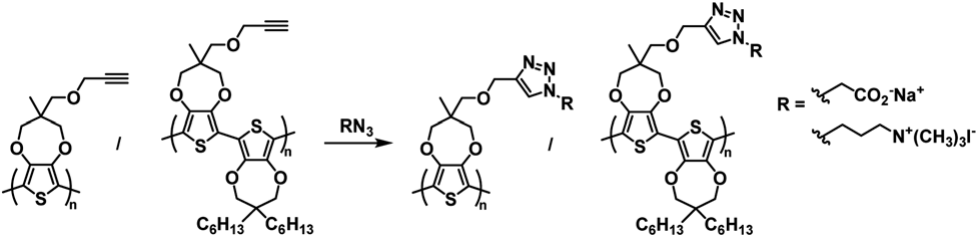
Functionalization *via* click chemistry of ProDOT-propargyl polymers to make water soluble polymers.^26^

One of the earliest examples of a specifically designed Type I electrochromic polythiophene comes from the Kumar group who in 2009 reported on an efficient way to make a variety of water soluble ProDOT-based polymers using click chemistry.^26^ By incorporating a propargyl substituent onto the ProDOT monomer, they were able to efficiently incorporate a variety of substituents through click chemistry, including hydrophilic carboxylate and ammonium substituents that made the polymers water soluble (Scheme 1). Polymers were synthesized through oxidative coupling with FeCl_3_. Copolymers of ProDOT-propargyl and ProDOT-dihexyl were also synthesized in order to improve the polymer solubility and click reaction yields. Both homopolymers and copolymers with carboxylate and ammonium substituents were fully soluble in water. Despite successfully synthesizing water soluble polymers, only the ProDOT-propargyl polymer precursor was characterized by spectroelectrochemistry. The polymer was electropolymerized onto ITO-coated glass slides at 1.20 V to form dark blue films with *λ*_max_ at 580 nm. Switching between −1.00 V and 1.00 V changed the film from dark blue and opaque to colourless and transmissive with new absorption at 950 nm and an optical contrast of 75%. The switching time measured for 95% of transmittance change at 580 nm was found to be ∼0.5 s. Overall, this study showed that an insoluble polymer with propargyl substituents could be made water soluble through click chemistry by incorporating hydrophilic groups.

**Scheme 2:**
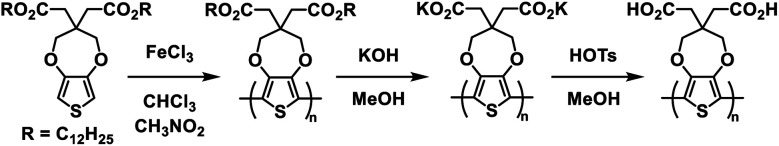
Synthesis of water processable and solvent resistant ProDOT polymers *via* side-chain defunctionalization.^27^

A wide variety of Type I ProDOT polymers have been reported by the Reynolds group over the years. In 2010 they introduced a water soluble ProDOT polymer prepared *via* side-chain defunctionalization (Scheme 2).^27^ The ProDOT polymer was prepared with dodecyl ester side groups and then it was subjected to ester saponification with potassium hydroxide in methanol to prepare the water soluble polymer with carboxylate groups. This polymer could be spray cast from 2 mg mL^−1^ aqueous solution onto ITO-coated glass slides to form films which were then submerged in 0.1 wt% *p*-toluenesulfonic acid in methanol to protonate the carboxylate groups and make films resistant to both organic and aqueous solvents. The acid film, purple in colour in the neutral form with absorption maxima at 576 nm and 625 nm, was redox cycled between −0.50 V and 0.55 V with oxidation leading to a fully transmissive and colourless film and an increase in NIR absorption. The optical contrast for the acid film was found to be about 60% with a switching rate from 10 s to 0.5 s. The films stability was also tested and it was found that 16 000 redox cycles with square wave potential steps of 1 s led to only a 5% loss in transmittance contrast. This study demonstrated a simple and efficient method of synthesizing sustainable water soluble polymers and using them to prepare solvent resistant films for optoelectronic device application.

**Scheme 3:**
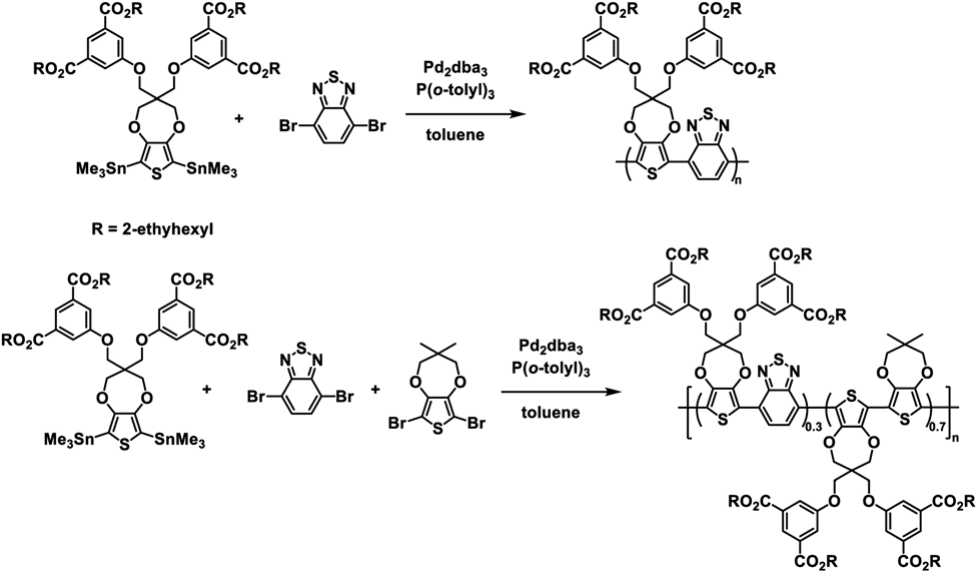
Synthesis of water processable and solvent resistant alternating and random ProDOT copolymers.^28^

The Reynolds group continued with their ester defunctionalization method of preparing water soluble polymers and in 2012 reported on two new polymers: one a donor–acceptor alternating copolymer and the other a donor acceptor random copolymer with ProDOT as the donor and benzothiadiazole (BT) as the acceptor.^28^ The ProDOT monomer was functionalized with four 2-ethylhexyl ester groups and combined *via* Stille coupling with benzothiadiazole to make the alternating copolymer and benzothiadiazole and dimethyl-ProDOT to make the random copolymer (Scheme 3). These ester copolymers were then subjected to the same ester defunctionalization process as the previous study^27^ to yield the water soluble carboxylate copolymers which could be spray cast onto ITO-coated glass slides and acidified to make solvent resistant films. The higher number of carboxylate functional groups allowed for higher water solubility of 4 mg mL^−1^ compared to the previous polymer.^27^

**Fig. 6:**
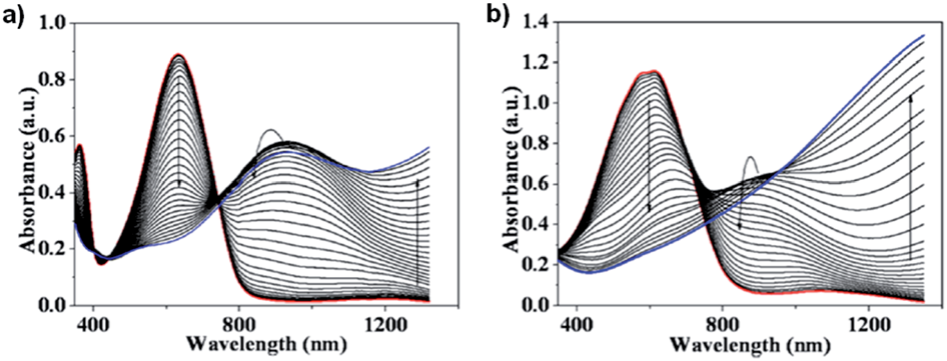
Spectroelectrochemistry of (a) alternating ProDOT/BT acid copolymer oxidized from 0 V to 0.8 V and (b) random ProDOT/BT acid copolymer oxidized from −0.2 V to 0.5 V. Adapted with permission from P. Shi, C. M. Amb, A. L. Dyer and J. R. Reynolds, *ACS Appl. Mater. Interfaces*, 2012, **4**, 6512–6521. Copyright 2012 American Chemical Society.

The optical properties of the water soluble copolymers showed absorption from 350 nm to 400 nm and 450 nm to 700 nm with a *λ*_max_ at 635 nm in solution and solid-state for the alternating copolymer, appearing greenish blue, and broad absorption from 400 nm to 750 nm with *λ*_max_ at 630 nm in solution and 555 nm in solid-state for the random copolymer, appearing dark blue. The acid films, which showed the same optical properties as their salt counterparts, were analysed by spectroelectrochemistry (Fig. 6). The alternating copolymer was oxidized from 0 V up to 0.8 V while the random copolymer oxidized at a lower potential from −0.2 V up to 0.5 V. The difference in oxidation onset potential between the alternating and random copolymers was attributed to the increased donor unit in the random copolymer leading to a higher ionization potential. Oxidation of both copolymers led to a loss of absorption in the visible region and the emergence of absorption in the NIR from 800 nm to 1200 nm. The electrochromic data for both polymers is shown in Table 2. The alternating copolymer had an optical contrast at 635 nm of 40% at 10 s switching time which remained stable with only minor decreases in optical contrast up to 0.5 s switching time. The random copolymer had a higher optical contrast at 555 nm of 52% at 10 s switching time remaining stable up to 1 s switching time. The switching times required to achieve 95% of the transmittance change were also determined for each polymer acid film. It was found that the alternating copolymer had a bleaching time of 1.06 s and colouration time of 0.19 s while the random copolymer had a shorter bleaching time of 0.44 s and longer colouration time of 0.39 s. This data is summarized in Table 2. Overall, this study demonstrated the synthesis of two new aqueous-processable electrochromic polymers with high water solubility and very fast electrochromic switching times.

**Table 2:** The electrochromic data for alternating and random ProDOT polymers. OCs were determined with oxidation at 0.8 V for the alternating copolymer and 0.5 V for the random copolymer. Response times were determined at 95% transmittance change^28^

Polymer	Wavelength (nm)	OC (%)	Response times (s)	
			Bleaching	Colouration
Alternating	635	40	1.06	0.19
Random	555	52	0.44	0.39

**Scheme 4:**
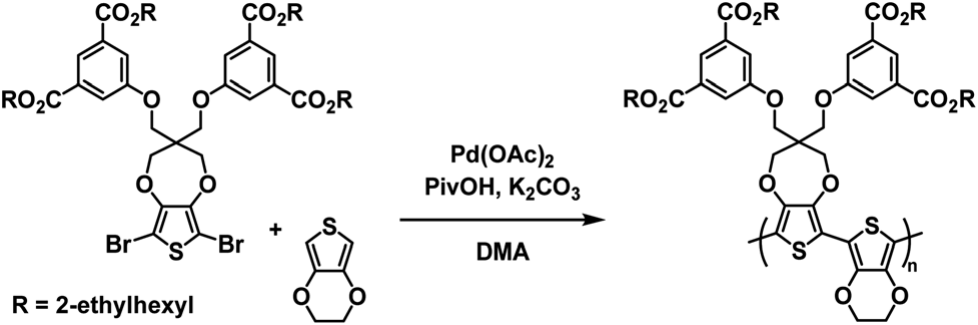
DHA synthesis of water processable and solvent resistant alternating ProDOT/EDOT copolymers.^29^

The Reynolds group would follow up on these results by preparing an alternating copolymer from the same ester-functionalized ProDOT monomer and EDOT to make poly(ProDOT-*alt*-EDOT) (PPE).^29^ This polymer was synthesized *via* direct (hetero)arylation polymerization (DHAP) methods, which involve the direct coupling of an aryl-bromide and aryl-H without the need for transmetalation reagents as in Stille or Suzuki coupling (Scheme 4).^57–59^ The ester polymer was produced in an excellent 94.4% yield.

Once again, the ester groups were hydrolyzed with potassium hydroxide to make an aqueous-processable polymer which was spray-cast onto ITO-coated glass and acidified to make a solvent resistant film. It was found that the solvent resistant films could be effectively electronically characterized in biologically compatible electrolyte solutions such as aqueous NaCl, human blood serum, and Ringer's solution which is compositionally similar to cerebral spinal fluid. This has important implications for application of electrochromic materials in medical research.

**Fig. 7:**
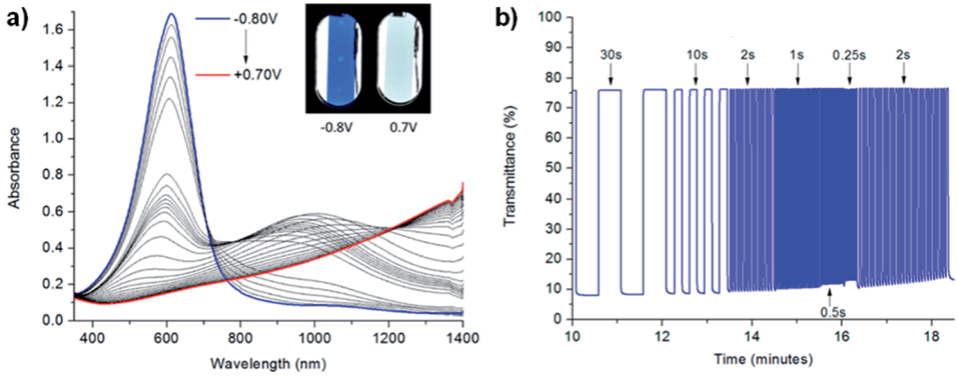
(a) Spectroelectrochemistry and (b) transmittance at *λ*_max_ as a function of switching time of spray-cast PPE acid film on ITO oxidized from −0.80 V to 0.70 V adapted with permission from J. F. Ponder, A. M. Österholm and J. R. Reynolds, *Chem. Mater.*, 2017, **29**, 4385–4392. Copyright 2017 American Chemical Society.

In the neutral state the acid film was blue with a *λ*_max_ of 613 nm and became colourless with increased NIR absorbance when oxidized up to 0.7 V (Fig. 7). Stepwise oxidation allowed the identification of the radical cation species with the polaronic absorption band at ∼1000 nm and the dication species with the bipolaron absorption band at >1500 nm. The films achieved high optical contrast of 68.0% at 613 nm with an exceptionally fast bleaching time achieving 95% transmittance change in 0.17 s (Fig. 7), much faster than has previously been observed. The fast switching time was attributed to the high ionic conductance of the saltwater electrolyte and the incorporation of EDOT units in the polymer backbone.

**Scheme 5:**
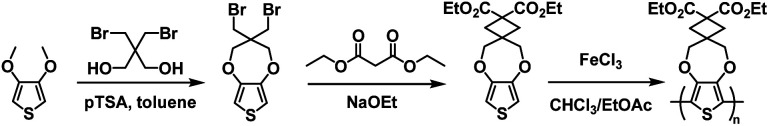
Synthesis of the water processable regiosymmetric ProDOT polymers.^30^

Inspired by the success of the Reynolds group's ester defunctionalization approach to producing water-processable polymers, the Xu group in 2015 reported on a highly regiosymmetric ProDOT polymer synthesized with a diethyl malonate functional group for solubility purposes connected to the ProDOT backbone *via* a cyclobutane spacer.^30^ The ProDOT monomer was synthesized *via* the transetherification of 3,4-dimethoxythiophene and 2,2-bis(bromomethyl) propane-1,3-diol followed by double nucleophilic substitution with diethyl malonate^60^ and the polymer was synthesized *via* oxidative coupling with FeCl_3_ and partially hydrolysed with sodium hydroxide (Scheme 5). This partially hydrolysed polymer was soluble in water up to 8 mg mL^−1^ and was spray-cast onto ITO-coated glass slides to form films which were then acidified to make solvent resistant films. The films were bright blue with two *λ*_max_ at 580 nm and 630 nm due to the high symmetry and rigidity in the ProDOT unit. Oxidizing from −0.15 V to 0.5 V resulted in loss of absorption in the visible spectrum and an increase in NIR absorption with *λ*_max_ at 950 nm. The polymer was also subject to colourimetry analysis and at −0.15 V the L*a*b* coordinates were found to be 50.1, 9.5, −51.8 corresponding to bright blue with total transmittance of 13.5%. Full oxidation resulted in L*a*b* coordinates of 85.5, −2.1, −5.2 corresponding light sky blue. The optical contrast was found to be 56% for 10 s to 3 s switching time, decreasing to 45% for 1 s switching time. The bleaching time for 95% transmittance change at 580 nm was found to be 1.8 s. The polymer film was also found to be quite stable, with only 11% contrast loss over 11 000 cycles with 5 s switching time. Overall, high water solubility, high regiosymmetry, and high optical contrast make this polymer very promising for ECD application.

### 3.2. Viologens

**Fig. 8:**
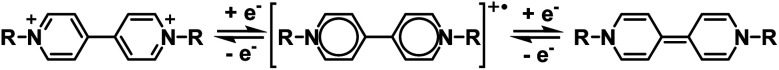
The oxidation states of viologens.

Viologens, or 4,4′-bipyridiliums, are dications that can be reversibly reduced twice to form a radical cation and neutral species (Fig. 8). The radical cation is relatively stable due to delocalization of the radical throughout the π system.^14^ This makes viologens particularly useful as electron transfer mediators in batteries,^61^ sensors,^62^ and field effect transistors,^63^ among other applications. They are also used in herbicides, which is certainly a negative concerning environmentally sustainability. By far the most common use for viologens is in ECDs for each oxidation state typically possesses a unique colour, making viologens very effective as multichromic materials. The colours typically depend on the R groups, counterions, and solvent and can be readily tuned by variation of those. Water solubility can also be determined by the R groups and counterions. For example, 1,1′dimethyl-4′4-bipyridilium (methyl-viologen, MV^2+^), perhaps the simplest viologen, is soluble in many solvents including water. As a dication, methyl-viologen is colourless and as a radical cation it is blue in organic solvents and purple in water, meaning methyl-viologen is solvatochromic in addition to Type I electrochromic. This is due to the radical cation dimerizing in aqueous media.^64,65^ The dimer species is red which combines with the blue monomer species in which it is in equilibrium to give purple. Several innovations have been made over the years with viologens to change their colours and improve their electrochromic performance, examples of which will be discussed below.

**Fig. 9:**
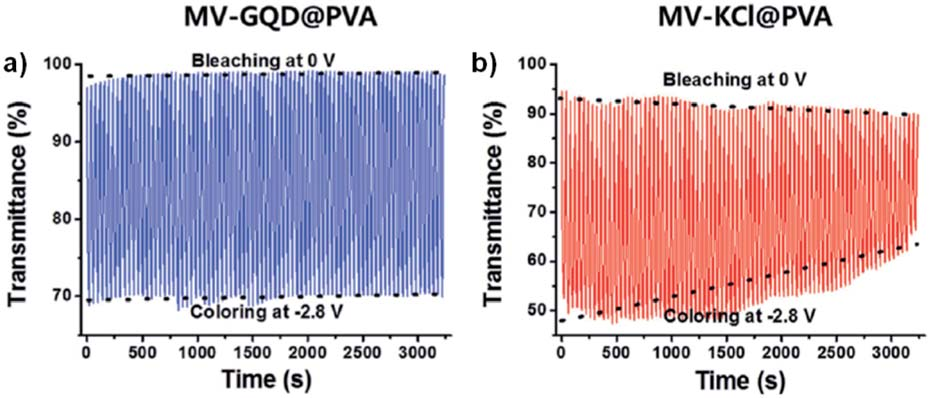
Voltage-controlled transmittance changes with switching between 0 V and −2.8 V for 50 mM MV^2+^ at 550 nm in (a) 8 mg mL^−1^ GQD in PVA and (b) 0.1 M KCl in PVA ECDs. Adapted with permission from E. Hwang, S. Seo, S. Bak, H. Lee, M. Min and H. Lee, *Adv. Mater.*, 2014, **26**, 5129–5136. Copyright 2014 John Wiley and Sons.

One example of using water soluble methyl-viologen in an ECD comes from the Lee group who in 2014 combined methyl-viologen dichloride with graphene quantum dots (GQDs) as cation/anion pairs in an electrolyte-free flexible ECD.^31^ The MV^2+^ and GQDs form strong intermolecular interactions through π–π stacking and electrostatic attractions *via* carboxylate groups in the GQDs. The ECD was fabricated by mixing MV^2+^ and GQDs in water and adding 10 wt% poly(vinyl alcohol) (PVA) solution to form a gel. This gel was then injected between two ITO-coated glass electrodes wrapped in Surlyn tape. The strong interaction between MV^2+^ and GQD was shown by GQD fluorescence quenching with increasing amounts of MV^2+^. The devices were colourless at 0 V and became purple when reduced at −2.8 V with a *λ*_max_ of 550 nm. This wavelength was monitored in spectroelectrochemical analysis to determine the electrochromic properties. The colour could be reversibly and repeatedly switched with potential cycling between 0 V (bleached) and −2.8 V (coloured). The MV^2+^/GQD devices were found to be more stable than electrolyte based MV^2+^/KCl devices with switching performance remaining stable over 3200 s with 20 s pulse widths (Fig. 9). The MV^2+^/GQD device maintained an optical contrast of 29% over this period while the MV^2+^/KCl device optical contrast decreased from 45% to 24%. The bleaching time, measured at 90% transmittance change, was 10 s for MV^2+^/GQD and 17 s for MV^2+^/KCl. Colouration efficiency was also higher at 65 cm^2^ C^−1^ for MV^2+^/GQD *vs.* 58 cm^2^ C^−1^ for MV^2+^/KCl. The stability of the MV^2+^/GQD device under harsh conditions was also tested by exposing the device to high temperatures. After sitting in an 80 °C oven for a few minutes the device was able to switch between coloured and bleached states and maintain an optical contrast of 20% over 2500 s. Flexible devices were also prepared by injecting the MV^2+^/GQD PVA gel between two ITO-coated polyethylene terephthalate (PET) electrodes. These devices were able to reliably switch between coloured and bleached states with colouration and bleaching times of 26 s and 39 s, respectively. The flexible devices were also able to perform when subject to bending stress with a slight decrease in optical contrast. Overall, this study demonstrated the first example of an electrolyte-free electrochromic carbon-based device that not only had improved electrochromic performance compared to the electrolyte-based analogue, but also could be made flexible which has important implications for various practical applications.

**Fig. 10:**
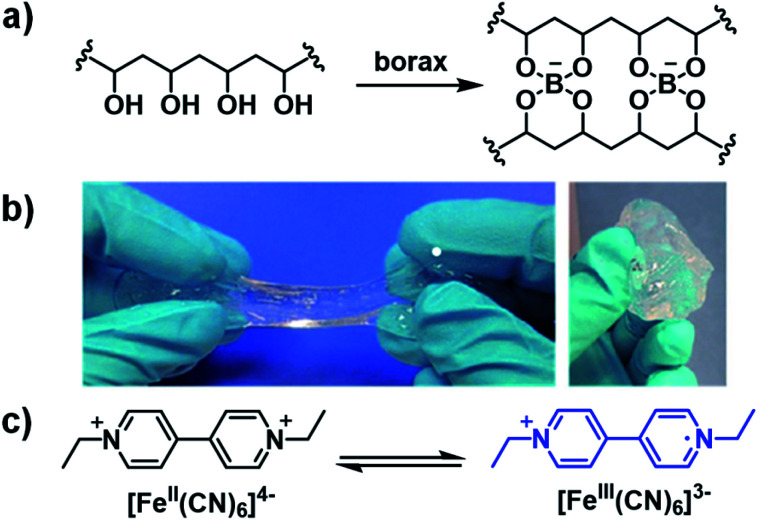
(a) Preparation of PVA/borax slime, (b) appearance of PVA/borax slime, and (c) ethyl viologen and ferrocyanide/ferricyanide redox pair used to fabricate the slime-based ECD. Adapted with permission from Y. Alesanco, J. Palenzuela, A. Viñuales, G. Cabañero, H. J. Grande and I. Odriozola, *ChemElectroChem*, 2015, **2**, 218–223. Copyright 2015 John Wiley and Sons.

Ethyl-viologen (EV^2+^) possesses the same electrochromic properties as methyl-viologen and has been applied in a slime-based ECD by Alesanco *et al.* in 2015.^32^ A slime-based electrolyte was desired as a substitute for solid-state electrolytes which often have defects as a result of their formation. The slime was prepared from a 4% PVA aqueous solution and 4% borax aqueous solution with various amounts of ethyl-viologen dibromide as the electrochrome and ferrocyanide/ferricyanide redox pair (1 : 1 K_4_[Fe(CN)_6_]/K_3_[Fe(CN)_6_]) as the redox mediator (Fig. 10). It was found that the optimum concentrations of electrochrome and redox pair were 20.0 mmol L^−1^ and 6.0 mmol L^−1^, respectively. The slime was then sandwiched between FTO-coated glass slides with 200 μm of adhesive tape as a spacer to make ECDs. The device was colourless at 0 V and violet at −2.3 V with a *λ*_max_ of 550 nm and optical contrast of 58%. Switching times, determined for 90% of transmittance change, were found to be 5 s and 4 s for colouration and bleaching, respectively, which were within the normal 1–8 s for liquid and gel-based electrolytes. The efficiencies were found to be 75.5 cm^2^ C^−1^ for colouration and 149.3 cm^2^ C^−1^ for bleaching. The much higher bleaching efficiency was ascribed to the ferricyanide mediator which is known to facilitate rapid oxidation of viologen radical cations. The CIE coordinates of the coloured state were also determined and were found to be L* = 7.12, a* = 18.08, and b* = −21.02, which corresponds to purple as a combination of red and blue according to the a* and b* values, respectively. As discussed above, the red and blue combination from the red viologen radical cation dimer, which forms in aqueous solutions, and blue radical cation monomer. Cyclability tests switching from −2.3 V for 10 s to 0 V for 180 s showed the device maintained OC at 58% up to 10 000 cycles with a decrease to 50% at 15 000 cycles and 33% at 25 000 cycles due to a slight pink colouration in the bleached state. This was likely a result of an irreversible reduction occurring due to electrochrome degradation. The slime was also characterized by rheology and it was found to possess a liquid-to-gel transition at 0.4 Hz, which was lower than the liquid-to-gel transition frequency for the pure PVA-borax slime at 2 Hz. This indicates the electrochromic slime possess more solid-like character which permits more facile manipulation of the electrolyte during device fabrication. Overall, this study demonstrated that high performance ECDs could be manufactured from a slime-based electrolyte with fast response times, high stability, and good cyclability.

**Fig. 11:**
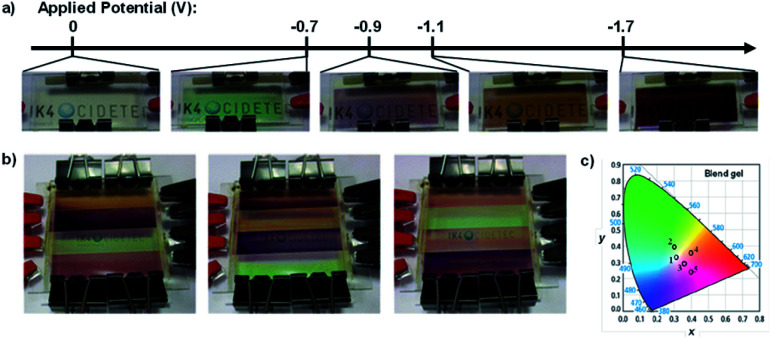
(a) Colours at various potentials for the electrochromic device fabricated from the blend gel of CPV^2+^ and EV^2+^. (b) Rainbow ECDs fabricated from blend gel of CPV^2+^ and EV^2+^. (c) CIE chromaticity plot indicating the colour coordinates for the rainbow ECD at 0 V (1), −0.7 V (2), −0.9 V (3), −1.1 V (4), and −1.7 V (5). Adapted with permission from Y. Alesanco, A. Viñuales, J. Palenzuela, I. Odriozola, G. Cabañero, J. Rodriguez and R. Tena-Zaera, *ACS Appl. Mater. Interfaces*, 2016, **8**, 14795–14801. Copyright 2016 American Chemical Society.

Viologen can of course be functionalized with more than just methyl and ethyl groups. Using more complex substituents can alter the viologen's optoelectronic properties and change the colours associated with the different oxidation states. In 2016 Alesanco *et al.* continued their research with PVA/borax-based ECDs by fabricating several multicoloured ECDs with *p*-cyanophenylviologen dichloride (CPV^2+^).^33^ The functionalization of viologen with *p*-cyanophenyl has significant impacts on the optoelectronic properties. Gels with CPV^2+^ were prepared by combining 4% PVA aqueous solution and 4% borax aqueous solution in a 4 : 1 volumetric ratio with the desired amounts of CPV^2+^ and 1 : 1 K_4_Fe(CN)_6_/K_3_Fe(CN)_6_. Devices were then fabricated by sandwiching the gel between ITO-coated glass slides. The CPV^2+^gel was colourless as the dication at 0 V; green as the radical cation at −0.5 V with absorption at 420 nm and 600 nm and L*a*b* CIE coordinates of 62, −44, 27; and reddish purple as the neutral species at −0.9 V with absorption at 500 nm and L*a*b* CIE coordinates of 29, 38, −15. The transition from colourless to green had an optical contrast of 61% and colouration efficiency of 78 cm^2^ C^−1^ and the transition from colourless to red had an optical contrast of 59% and colouration efficiency of 83 cm^2^ C^−1^. Switching times were also measured for each transition at 90% transmittance change. At −1.4 V colouration took 16 s and bleaching took 8 s and at −1.8 V colouration took 14 s and bleaching took 4 s. Because of the multichromic behaviour displayed by CPV^2+^ achieved at relatively low reduction potentials, a blend gel with both CPV^2+^ and EV^2+^ was used to fabricate a multi-coloured ECD. A cathodic sweep from 0 V to −1.7 V identified five reduction potentials associated with this device: −0.2 V for reduction of K_3_Fe(CN)_6_; −0.5 V and −0.9 V for the first and second reductions of CPV^2+^, respectively, and −1.2 V and −1.6 V for the first and second reductions of EV^2+^, respectively. As a result, this device had five coloured states: colourless at 0 V, green at −0.7 V (absorption bands at 420 nm and 600 nm), pinkish violet at −0.9 V (520 nm), orange at −1.1 V (460 nm), and purple at −1.7 V (520 nm) (Fig. 11a). A “rainbow” device was also fabricated by layering the blend gel in isolated rows that could have their potential adjusted independently (Fig. 11b) and the colour coordinates were plotted onto the CIE chromaticity diagram (Fig. 11c). The ease and simplicity with which their devices were fabricated has significant implications for the development of organic colour displays and smart windows.

**Scheme 6:**
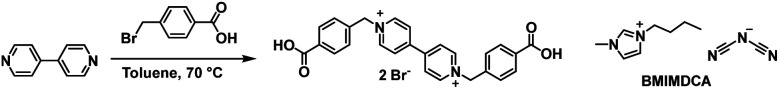
Synthesis of benzoic acid substituted viologen BAV^2+^ and components of ionic liquid 1-butyl-3-methylimidazolium dicyanamide (BMIMDCA).^34^

In 2015 Benedetti *et al.* reported on an all solid-state ECD based on a viologen functionalized with benzoic acid substituents (BAV^2+^, Scheme 6).^34^ The device was fabricated by incorporating BAV^2+^ in an ionogel, a gel formed from by encapsulating an ionic liquid in a solid matrix. Ionogels have been shown to possess interesting conductivity properties for electrochemical applications.^66–68^ In this study, the researchers used the ionic liquid 1-butyl-3-methylimidazolium dicyanamide (BMIMDCA, Scheme 6) with gelatin as the gelator. The electrochromic ionogel was formed by stirring 20 mg of BAV^2+^ and 60 mg of gelatin in 300 μL of BMIMDCA for 2 h at 40 °C followed by the addition of 500 μL of water at 40 °C to form a pale yellow gel. An even film of the gel was then spread onto an electrochemical cell prepared from an ITO-coated glass slides with silver and platinum electrodes. Spectroelectrochemical analysis indicated the gel could be reversibly reduced at −0.3 V to form BAV˙^+^ with a colour change to dark violet. Reduction to form neutral BAV could be achieved at −0.6 V but this process was found to be irreversible. Reversible reduction occurred up to −0.4 V which resulted in the most intense colour change with a broad absorption bands at 550 nm and 900 nm. The violet colour was once again a result of the combination of red radical cation dimer and blue radical cation monomer. The device stability was probed by cycling between −0.25 V and −0.4 V for 30 s and 0.8 V for 60 s. It was found that the device was stable for up to 75 cycles with reduction at −0.25 V than with reduction at −0.4 V (only 15 cycles of stability) but for both reduction potentials the negative current decreased over time while the positive current remained stable. This implied that irreversible reduction of BAV^2+^ was occurring even at the lower reduction potential, which was also accompanied by a smaller optical contrast. In conclusion, while this study did demonstrate that a solid-state ECD could be fabricated using environmentally friendly ionogel technology, stability issues would need to be addressed with further research.

**Fig. 12:**
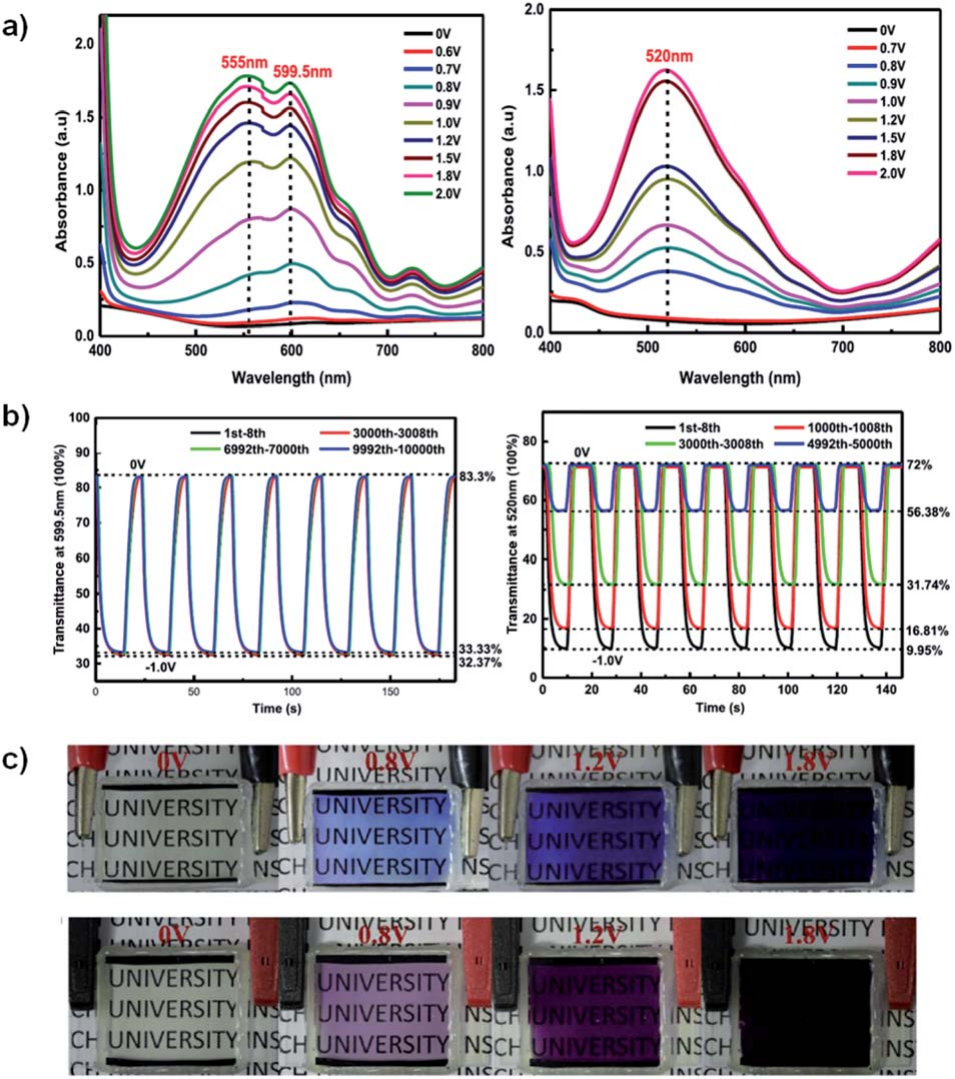
(a) Absorption profiles for PVB gel (left) and PVA gel (right) ECDs at various potentials. (b) Comparisons of transmittance changes *vs.* time upon cycling PVB gel ECD (bleached 0 V for 10 s and coloured −1.0 V for 15 s, left) and PVA gel ECD (bleached 0 V for 10 s and coloured −1.0 V for 10 s, right). (c) Images of PVB gel (top) and PVA gel (bottom) ECDs at various potentials. Reprinted from *Electrochim. Acta*, vol. **298**, S. Zhao, W. Huang, Z. Guan, B. Jin and D. Xiao, A novel bis(dihydroxypropyl) viologen-based all-in-one ECD with high cycling stability and coloration efficiency, pages 533–540, Copyright 2019, with permission from Elsevier.

A recent example of a water soluble viologen comes from the Xiao group who in 2019 reported on a viologen functionalized with dihydroxylpropyl substituents to improve ECD performance.^35^ This compound, DHPV^2+^, was synthesized as the dichloride salt from 4,4′-bipyridine and 3-chloro-1,2-propanediol in DMF and used to make two electrochromic gels, one based on poly(vinylbutyal) (PVB) in methanol and the other based on PVA and borax in water. Both gels were sandwiched between ITO-coated glass slides when in the flow state to fabricate ECDs. The electrochromic data for both devices is shown in Table 3. At 0 V both gels are colourless. The PVB gel begins to change colour at −0.7 V with absorption peaks appearing at 555 nm and 600 nm (Fig. 12a). These peaks increase in intensity up to −2 V at which point the gel is a dark blue from DHPV˙^+^ with an optical contrast of 51%. The PVA gel begins to change colour at −0.8 V with a peak appearing at 520 nm and increases in intensity up to −2 V at which point the gel is dark purple from DHPV˙^+^ monomer and dimer with a higher optical contrast of 62% (Fig. 12a). Cyclability and stability tests showed that the PVB gel was very stable maintaining 98% of the optical contrast over 10 000 cycles while the PVA gel was much less stable with optical contrast dropping to 25% of the original after only 5000 cycles (Fig. 12b). The PVB gel also had a higher colouration efficiency of 301 cm^2^ C^−1^ compared to 165 cm^2^ C^−1^ for the PVA gel. Switching times were also calculated for each gel, measured at 95% of the transmittance change. The PVB gel had colouration and bleach times of 5.6 s and 5.3 s, respectively, and the PVA gel had colouration and bleach times of 4.5 s and 2.1 s. Since the PVB had improved electrochromic properties over the PVA gel it was selected to fabricate a large area ECD that was 14 cm × 12 cm. This device was able to switch from bleached to coloured state in 12 s at a relatively low −1.5 V. Overall, this paper showed that a water soluble viologen could be incorporated in various gel matrices. The PVB methanol-based electrochromic gel outperformed the PVA/borax hydrogel in terms of reduction potential, stability, and colouration efficiency and was shown to even function in a large area electrochromic, an important step to develop ECDs for widescale use.

**Table 3:** Electrochromic data for PVB and PVA gels with DHPV^2+^ electrochrome. Response times were determined at 95% transmittance change^35^

Gel	*λ* (nm)	OC (%)	CE (cm^2^ C^−1^)	Response times (s)	
				Colouration	Bleaching
PVB	555, 600	51	301	5.6	5.3
PVA	520	62	165	4.5	2.1

**Scheme 7:**
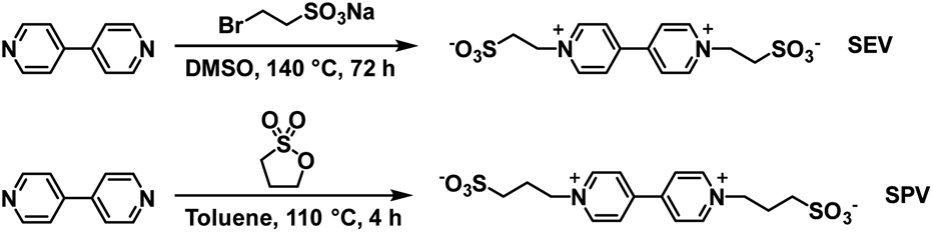
The synthesis of viologens SEV and SPV.^36^

The Xiao group has continued their research with water soluble viologens and has recently reported on two viologens functionalized with sulfonate groups which removes the need for additional electrolytes.^36^ These are 1,10-bis(2-sulfonatoethyl) viologen (SEV) and 1,10-bis(3-sulfonatopropyl)viologen (SPV) and their syntheses are shown in Scheme 7.

**Fig. 13:**
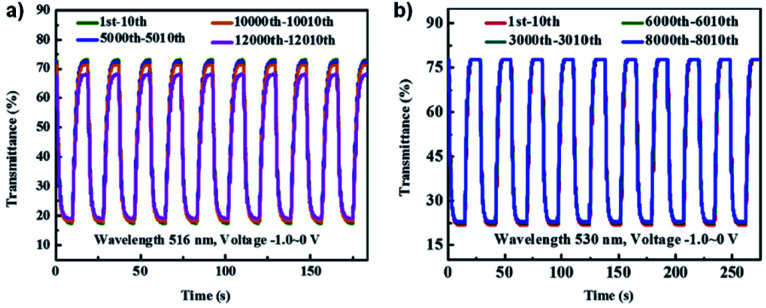
Comparison of transmittance changes *vs.* time for (a) SEV hydrogel ECD at 516 nm cycling 0 V to −1.0 V for 10 s and (b) SPV hydrogel ECD at 530 nm cycling 0 V to −1.0 V for 15 s. Reprinted from *Dyes Pigm.*, vol. **174**, S. Xiao, Y. Zhang, L. Ma, S. Zhao, N. Wu and D. Xiao, Easy-to-make sulfonatoalkyl viologen/sodium carboxymethylcellulose hydrogel-based electrochromic devices with high coloration efficiency, fast response and excellent cycling stability, page 108055, Copyright 2019, with permission from Elsevier.

These viologens were used to fabricate electrochromic hydrogels with 1% SEV or SPV as the electrochrome, 0.5% 1,1′-ferrocenedimethanol as the complementary redox species, 8.5% sodium carboxymethylcellulose (CMC-Na) as the conductive gelator, and 90% water. These gels were then sandwiched between ITO-coated glass to prepare ECDs. Electrochromic data is shown in Table 4. At 0 V the gels were transparent pale yellow. Reduction at −0.8 V led to absorption peaks appearing at 516 nm and 895 nm for the SEV gel and 530 nm and 940 nm for the SPV gel and increasing in intensity up to −2.2 V. These changes were associated with the formation of the radical cations of both viologens with SEV gel becoming rose red (OC 55.75%) and SPV gel becoming purple (OC 55.98%). Cycling between 0 V and −1.0 V at 10 s intervals showed that the SEV gel only lost 2% of OC after 10 000 cycles and 6.56% after 12 000 cycles while the SPV gel lost 1% of OC after 8000 cycles (Fig. 13). Both were more stable than previously reported PVA/borax-based hydrogels. The switching times were determined for 90% of the transmittance change. The SEV gel had colouration and bleaching times of 2.8 s and 3.9 s at 516 nm, respectively, and 4.8 s and 3.7 s at 895 nm, respectively. The SPV gel had colouration and bleaching times of 3.1 s and 4.0 s at 530 nm, respectively, and 4.1 s and 2.4 s at 940 nm, respectively. These switching times are also shorter than many previously reported for viologen-based ECDs. The CEs were also determined for both hydrogels scanned between 0 V and −1.0 V. The SEV gel had colouration efficiencies of 296 cm^2^ C^−1^ and 74 cm^2^ C^−1^ at 516 nm and 895 nm, respectively, and the SPV gel had CEs of 304 cm^2^ C^−1^ and 72 cm^2^ C^−1^ at 530 nm and 940 nm, respectively. Again, these are higher than many previously reported viologen-based ECDs including that for the Xiao groups previous DHPV^2+^.^35^ Overall, this report demonstrates that viologen-based hydrogels can be used to fabricate high performance ECDs with high stability, fast response times, and high CE into the NIR.

**Table 4:** Electrochromic data for SEV and SPV. Response times were determined at 90% transmittance change^35^

Material	*λ* (nm)	OC (%)	CE (cm^2^ C^−1^)	Response times (s)	
				Colouration	Bleaching
SEV	516	55.75	296	2.8	3.9
	895	—	74	4.8	3.7
SPV	530	55.98	304	3.1	4.0
	940	—	72	4.1	2.4

### 3.3. Naphthalene diimides

Given the high toxicity of viologens, it is preferrable to use a safer compound in water processable ECDs, such as naphthalene diimide (NDI). Naphthalene diimides, along with the related compounds perylene bisimide (PBIs), are commonly used as n-type materials in a wide variety of organic electronic devices including solar cells,^69,70^ field effect transistors,^71,72^ light emitting diodes,^73,74^ and ECDs.^75,76^ They possess high electron affinity and extensive π-systems that provide stability for radical species and as such can be readily reduced. Generation of a radical anion *via* reduction also often results in a change in absorption profile. NDIs and PBIs can also be functionalized to tune their optoelectronic and solubility properties.^71,77,78^ NDIs typically possess Type I electrochromism while PBIs possess Type II electrochromism. Unlike many viologens, NDIs and PBIs are not inherently water soluble and must be made so through solubilizing functional groups. There are many examples of water-soluble NDIs and PBIs, including ones used in ECDs.

**Scheme 8:**
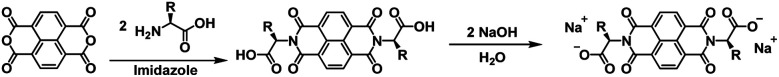
Synthesis of water soluble NDIs with amino acids.

Our group's interest in this area comes from our research in developing water soluble NDIs. Our strategy involves incorporating amino acids at the imide positions to synthesize water soluble NDIs (Scheme 8). The carboxylic acid moieties of the amino acids can be readily deprotonated to make fully water soluble NDIs. Combining this with NDI's electrochromic properties allows us to develop unique water soluble materials for ECDs.

**Fig. 14:**
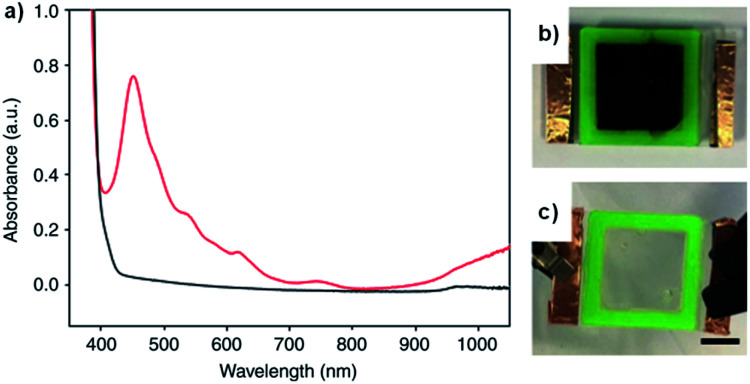
(a) The absorption profile for the NDI-GF gel irradiated with UV light (red) and after applying 0.6 V for 60 s (black). The electrochromic gel window (b) irradiated with UV light and (c) after applying 0.6 V for 60 s. Adapted with permission from L. Gonzalez, C. Liu, B. Dietrich, H. Su, S. Sproules, H. Cui, D. Honecker, D. J. Adams and E. R. Draper, *Commun. Chem.*, 2018, **1**, 1–8.

We recently reported on a water soluble NDI functionalized with the dipeptide glycine-phenylalanine (NDI-GF) that possesses unique transparent to black electrochromism.^37^ Colourless to black electrochromism is highly desirable for its application in smart windows for energy efficiency and privacy uses. Using this particular dipeptide enabled the material to form gels in water and 80 : 20 water/glycerol solutions. As discussed above, there are many examples of electrochromes being incorporated in gel electrolytes but, to the best of our knowledge, this is the first report of the gel matrix itself being the electrochrome. NDI-GF was fully dissolved in the aqueous solvent with two equivalents of NaOH and then the solution was gradually acidified by the slow hydrolysis of glucono-*δ*-lactone. This causes the NDI-GF molecules to self-assemble into a fibrillar network that entraps the solvent and forms the gel. The gels are initially colourless or pale yellow with strong absorption below 400 nm. Irradiation at 365 nm causes the gels to turn dark with *λ*_max_ at 460 nm, indicating the formation of a radical anion (Fig. 14a). Despite not having pan-chromatic absorption, the gels appeared black at high NDI-GF concentration. This colour change can also be induced electrochemically with reduction at −0.7 V. The colour change can be reversed upon oxidation at 0.6 V or with exposure to air. Cycling between transparent and black states could be achieved up to 100 cycles with little variation in the optical properties. Kinetic studies indicated the colouration time was faster than the switching time. The electrochromic gel was sandwiched between FTO-coated glass to make ECDs that could be switched either by electrochemical reduction or irradiation (Fig. 14b). Using masks allowed patterns to be created in the gels upon irradiation which retained their shape owing to the immobile nature of the gels. Overall, this study demonstrated the potential for water soluble organic electrochromes to achieve highly desirable transparent to black electrochromism, an important development for the application of such systems in smart windows.

## 4. Type II: coloured to coloured materials

Type II electrochromic materials are those that switch between differently coloured states and do not possess a colourless transparent state. As such, they are typically not used in smart window technology and are more often employed as sensors or in display technology, where they face considerable competition from OLEDs. Because they do not possess a colourless transparent state, characterization methods such as optical contrast and colouration efficiency are less applicable but are still often employed in reference to specific wavelengths in the optical spectra. More often, Type II electrochromic materials are characterized by colourimetry, whereby the colours of each oxidation state are quantified following the CIE colour space standards (Fig. 2). Like Type I materials, water soluble Type II materials also come in polymeric and molecular forms. Polymeric examples include polythiophene derivatives and polyaniline (PANI) and molecular examples primarily consist of PBIs.

### 4.1. Type II polythiophene derivatives

As discussed above, polythiophene derivatives possess remarkable optoelectronic properties which makes them ubiquitous in organic electronics research. Polythiophene itself is a Type II electrochromic material, possessing red to blue electrochromism upon oxidation.^22,55^ Its derivatives such as PEDOT and PProDOT are Type I with and are commonly much use in ECDs owing to their colourless to blue electrochromism. Further functionalization of PEDOT can alter its optoelectronic properties and turn it into a Type II electrochrome. Examples of Type II polythiophene derivatives, including PEDOT variants, are discussed below.

**Scheme 9:**
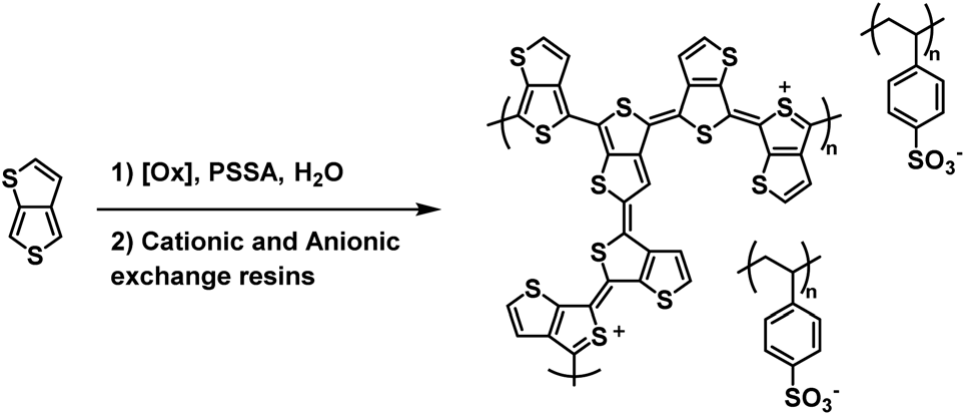
Synthesis of the water soluble PT34bT/PSS.^38^

One of the earliest examples of a water soluble Type II electrochromic polythiophene derivative comes from the Sotzing group who in 2005 reported on a water soluble polymer based on thieno[3,4-*b*]thiophene.^38^ This polymer, PT34bT, was prepared in water using a variety of chemical oxidants in the presence of PSSA to form PT34bT/PSS colloidal dispersions (Scheme 9). Because T34bT has three α positions to the S heteroatoms, branched polymers were formed. It was found that using chemical oxidants like ammonium persulfate and hydrogen peroxide improved the stability of the dispersions. These oxidants generate sulfate and hydroxyl radicals which were presumed to terminate propagation and incorporate onto the polymer backbone by radical coupling, thereby increasing hydrophilicity and improving stability in aqueous media. Upon polymerization the aqueous dispersions were purified by passing through cationic and anionic exchange resins. The type of chemical oxidant used in the polymerization affected the dispersions' optical properties. For example, using ammonium persulfate/ferric sulfate mixture resulted in broad absorption in the Vis/NIR absorption spectrum with a *λ*_max_ of 1080 nm in the oxidized form and 760 nm in the reduced form while using just ferric sulfate hydrate resulted in a *λ*_max_ of 1350 nm in the oxidized form and 910 nm in the reduced form, bathochromic shifts of 270 nm and 160 nm, respectively. All polymer dispersions were found to be transmissive green in the oxidized form and transmissive blue in the reduced form. Conductivities also varied depending on chemical oxidant, averaging *ca.* 10^−3^–10^−4^ S cm^−1^. Overall, this study expanded the range of water soluble polythiophene derivative colloidal dispersions to include Type II electrochromic materials, beyond the Type I PEDOT/PSS water soluble colloidal dispersion that had primarily been studied.

**Scheme 10:**
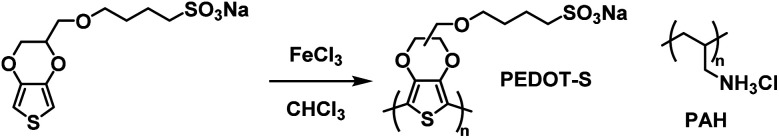
Synthesis of PEDOT-S *via* oxidative coupling and PAH copolymer.^39^

An early example of a Type II water soluble PEDOT used in an ECD was reported in 2002 by the Reynolds groups.^39^ They incorporated a sulfonate functional group onto the backbone to make PEDOT-S, a fully water soluble PEDOT without the need for PSS to make water soluble dispersions. The polymer was synthesized from the EDOT-S monomer *via* oxidative coupling with FeCl_3_ (Scheme 10). In the neutral state the polymer is dark purple with absorbance *λ*_max_ at 530 nm which changes to dark brown when partially oxidized and light blue when fully oxidized with absorption in the NIR (*λ*_max_ at 850 nm). Films with alternating layers of PEDOT-S and polyaniline hydrochloride (PAH) were prepared by sequentially casting onto ITO-coated glass slides from aqueous solutions pH adjusted to ∼2.8 with HClO_4_.^39^ It was found that alternating layers of PEDOT-S and PAH led to improved film homogeneity with self-healing characteristics. Using this method, the Reynolds group was able to reproducibly cast films with even thickness for up to 40 bilayers. Cycling between 0.6 V and −0.8 V led to reversible colour change from light blue to pink/purple with a 45% change in transmittance between the two states for a 40-bilayer film. Thinner films of 10 to 20 bilayers fully switched in 2.5 seconds, faster than thicker films of 30 to 40 bilayers which achieved 82% and 76% of the change in 2.5 seconds, respectively. The thinnest 10 bilayer film had the highest CE of 434 cm^2^ C^−1^ which decreased to 200–250 cm^2^ C^−1^ for thicker films. In a following study it was reported that solutions and films of PEDOT-S changed colour from pink to blue upon addition of an acid such as *p*-tolunesulfonic acid, hydrochloric acid, or perchloric acid, and returned to pink upon neutralization with hydrazine or sodium hydroxide.^79^ It was also found that addition of acid led to an increase in film conductivity which, combined with the colour change, implied that the same electronic changes were occurring in PEDOT-S as when the polymer was electronically or chemically oxidized. Overall, the Reynolds group found that though PEDOT-S/PAH films display rapid colour changes when electrochemically initiated, the contrast is lower than other electrochromic polymers and that the polymer would be better suited in sensors, indicators, or OLEDs than in strictly electrochromic devices.^79^

**Scheme 11:**
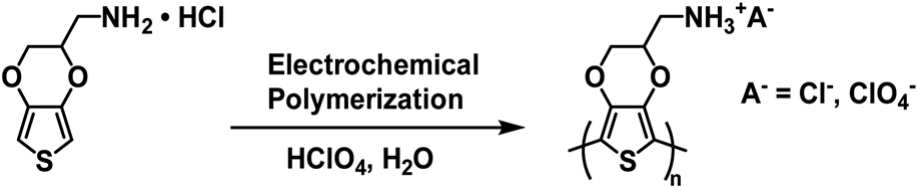
Synthesis of PEDOT-CH_2_NH_3_^+^A^−^*via* electrochemical polymerization.^40^

In 2016 the Duan and Xu groups reported on a Type II water soluble PEDOT polymer synthesized from EDOT bearing a methylamine hydrochloride side group (EDOT-CH_2_NH_2_·HCl).^40^ The polymer was synthesized *via* electrochemical polymerizing using 0.01 M monomer in 1 M HClO_4_ aqueous solution (Scheme 11). Films of PEDOT-CH_2_NH_3_^+^A^−^ (where A^−^ = Cl^−^ and ClO_4_^−^) and PEDOT-CH_2_NH_3_^+^ClO_4_^−^ were prepared by electrodepositing onto ITO-coated glass slides.

**Fig. 15:**
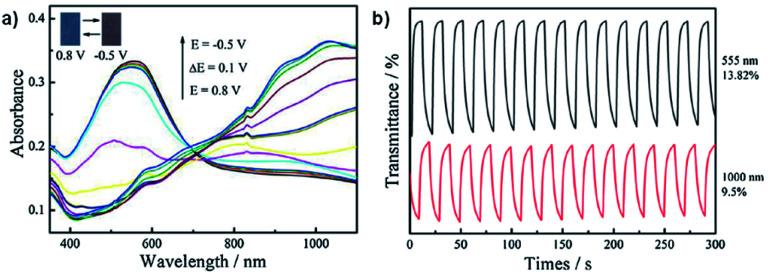
(a) Spectroelectrochemistry of PEDOT-CH_2_NH_3_^+^ClO_4_^−^ films on ITO-coated glass oxidized from −0.5 V to 0.8 V. (b) Transmittance as a function of switching time for PEDOT-CH_2_NH_3_^+^ClO_4_^−^ with potential oscillating between −0.5 V and 0.8 V with 10 s interval. Reprinted from *Synth. Met.*, vol. **211**, H. Sun, L. Zhang, L. Dong, X. Zhu, S. Ming, Y. Zhang, H. Xing, X. Duan, and J. Xu, Aqueous electrosynthesis of an electrochromic material based water-soluble EDOT-MeNH_2_ hydrochloride, pages 147–154, copyright 2016, with permission from Elsevier.

Neutral films of PEDOT-CH_2_NH_3_^+^A^−^ and PEDOT-CH_2_NH_3_^+^ClO_4_^−^ are reddish in colour with *λ*_max_ at 603 nm and 555 nm, respectively. Stepwise oxidation up to 0.8 V at 0.1 V steps results in a loss of absorption at 603 nm and 555 nm and increased absorption >730 nm with a colour change to blue. The OC, CE, and response times were determined for both films by cycling between −0.5 V and 0.8 V with 10 s switching time. Data is displayed in Table 5. The optical contrast of PEDOT-CH_2_NH_3_^+^A^−^ was found to be 26.38% at 603 nm and 17.47% at 1100 nm with CEs of 156 cm^2^ C^−1^ and 55 cm^2^ C^−1^, respectively, while the optical contrast of PEDOT-CH_2_NH_3_^+^ClO_4_^-^ was found to be 13.82% at 555 nm and 9.5% at 1000 nm with CEs of 31 cm^2^ C^−1^ and 8 cm^2^ C^−1^, respectively. Response times measured at 95% of the transmittance change for PEDOT-CH_2_NH_3_^+^A^−^ were found to be 1.4 s for oxidation and 9.6 s for reduction at 603 nm and 3.2 s for oxidation and 6.0 s for reduction at 1100 nm while response times for PEDOT-CH_2_NH_3_^+^ClO_4_^−^ were found to be 4.4 s for oxidation and 7.8 s for reduction at 555 nm and 8.0 s for oxidation and 7.2 s for reduction at 1000 nm. Overall, this study showed that the water soluble polymer PEDOT-CH_2_NH_3_^+^A^−^ could be more easily synthesized and possessed improved electrochromic properties compared to PEDOT-CH_2_NH_2_ and PEDOT (Fig. 15).

**Table 5:** The electrochromic data for PEDOT-CH_2_NH_3_^+^A^−^, PEDOT-CH_2_NH_3_^+^ClO_4_^−^, and PEDOT. OC, CE, and switching times were determined by cycling between −0.5 V and 0.8 V with 10 s switching time. Response times were recorded at 95% transmittance change^40^

Material	*λ* (nm)	OC (%)	CE (cm^2^ C^−1^)	Response times (s)	
				Colouration	Bleaching
PEDOT-CH_2_NH_3_^+^A^−^	603	26.38	156	1.4	9.6
	1100	17.47	55	3.2	6.0
PEDOT-CH_2_NH_3_^+^ClO_4_^−^	555	13.82	31	4.4	7.8
	1000	9.5	8	8.0	7.2
PEDOT	585	54	137	0.36	—

### 4.2. Polyaniline derivatives

**Fig. 16:**
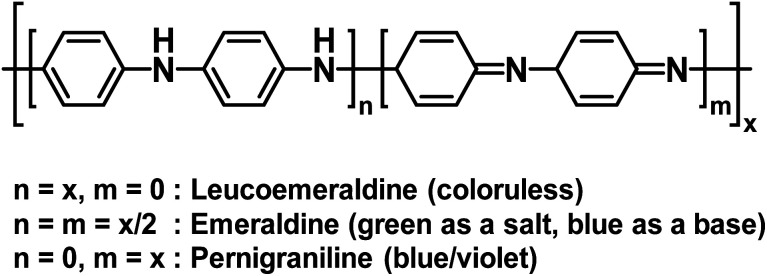
The structure and oxidation states of PANI.

Polyaniline (PANI) is an electrochromic polymer first reported in 1834 and synthesized primarily by oxidative polymerization of aniline.^41^ The polymer has three idealized oxidation states each with their own name and colour (Fig. 16). These are leucoemeraldine, emeraldine, and pernigraniline. Leucoemeraldine is the fully reduced state of the polymer and is colourless. Emeraldine is an intermediate state between fully reduced and fully oxidized and is green under acidic conditions and blue under basic conditions. Pernigraniline is the fully oxidized state and is blue violet. Despite being classified as a conductive polymer, none of these states are conductive by themselves. Only when the partially oxidized emeraldine is protonated at the imine positions are charge carriers generated and the polymer becomes conductive.^41^ In this form, PANI is not soluble in organic solvents and only soluble in acidic aqueous solvents and as such it is often paired with PSSA to form the PANI/PSS salt, much like PEDOT/PSS dispersions. Thus, because PANI is already electrochromic and water soluble by itself, most electrochromic research involving PANI is focused more on improving PANI's conductivity and performance in electronic devices rather than modifying the polymer backbone, as was the case for the polythiophene derivatives shown above. Despite possessing a colourless state, the idealized fully reduced leucoemeraldine form is rarely used and most PANI-based ECDs utilize the emeraldine and pernigraniline forms making PANI a Type II electrochromic material in practice.

**Fig. 17:**
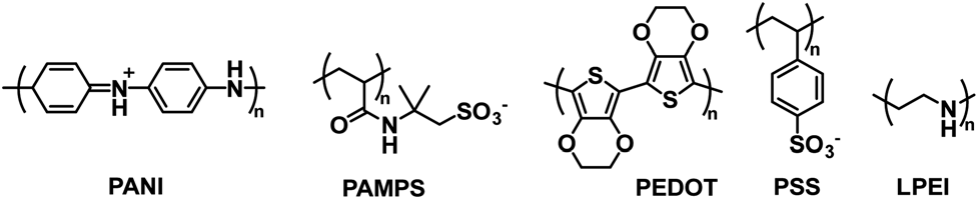
The water soluble polymers employed by the Hammond group to fabricate electrochromic cells.^42^

One early example of PANI incorporation in ECDs with no polymer modification comes from the Hammond group who in 2001 combined PANI with PEDOT/PSS in a layer-by-layer ECD architecture.^42^ They used “off the shelf” commercially available PANI and PEDOT/PSS without any modification to demonstrate the effectiveness of the layer-by-layer fabrication method for ECDs. In device construction, PANI was combined with the polyanion poly(2-acrylamido-2-methylpropanesulfonate) (PAMPS) and PEDOT/PSS was combined with the polycation linear poly(ethyleneimine) (LPEI) (Fig. 17). As discussed above, PANI is colourless when fully reduced and coloured when partially or fully oxidized while PEDOT is coloured when reduced and colourless when oxidized, so their combination would result in complementary multicoloured ECDs. Electrochromic cells were constructed with 20 or 40 bilayers in the configuration (PANI/PAMPS)_*x*_|(PAMPS·H_2_O)|(PEDOT/PSS/LPEI)_*x*_ where *x* = the number of bilayers, referred to as Cell_20_ and Cell_40_. The devices operate whereby oxidation of one electrochromic material results in reduction of the other with protons shuttling between PSS and PAMPS. The cells cycled between blue/green when oxidized and yellow when reduced with the colour primarily influenced by the strongly absorbing PANI. Cell_20_ had an optical contrast of 24% while Cell_40_ had an optical contrast of 30%. The stability of Cell_20_ was maintained over 35 000 switching cycles though variation in optical contrast was not measured. The switching time was defined as the time required to achieve 75% of the transmittance change. Cell_20_ had a bleaching time of 0.37 s and colouration time of 1.22 s while Cell_40_ had a bleaching time of 0.66 s and colouration time of 1.77 s. In summary, this study was an early example of how multicoloured ECDs could be fabricated in aqueous media with unmodified commercially available polymers.

**Scheme 12:**
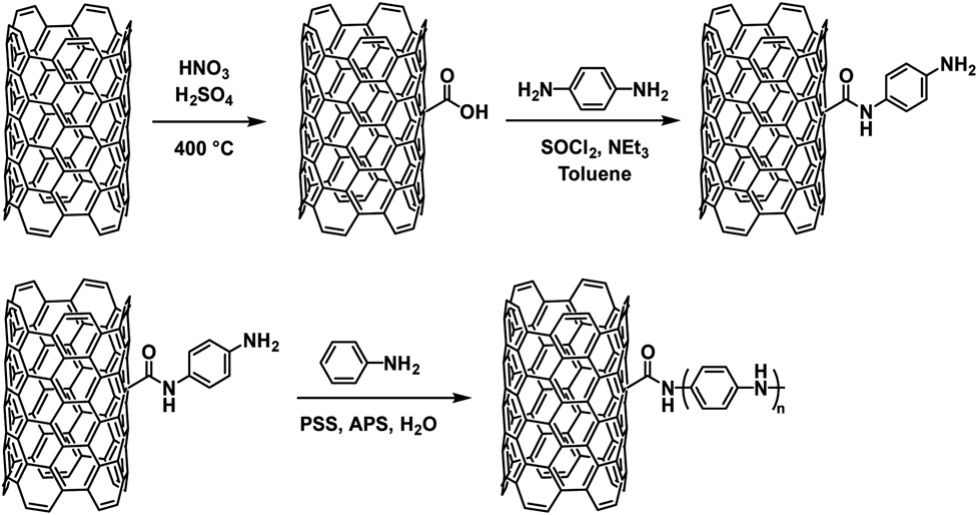
The synthesis of SWCNTs functionalized with PANI.^43^

Another example of PANI's use in ECDs comes from Xiong *et al.* who grafted PANI onto single-walled carbon nanotubes (SWCNTs).^43^ To do this, they oxidized SWCNTs with HNO_3_/H_2_SO_4_ to add carboxylic acid groups on the surface and then coupled with *p*-phenylenediamine (Scheme 12). This provided an anchor for polymerization with aniline which was performed in water with PSS as a dopant and ammonium peroxydisulfate (APS) as the oxidant resulting in SWCNTs enwrapped in PANI chains.

**Fig. 18:**
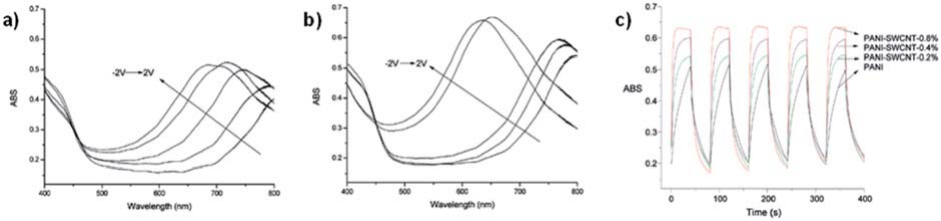
The spectroelectrochemistry of (a) PANI and (b) PANI-SWCNT-0.8% oxidized from −2 V to 2 V. (c) Optical absorbance at *λ*_max_ for devices with PANI, PANI-SWCNT-0.2%, PANI-SWCNT-0.4%, and PANI-SWCNT-0.8% as the electrochromic layers with potential oscillating between 2.0 V and −2.8 V with a 40 s interval. Adapted with permission from S. Xiong, J. Wei, P. Jia, L. Yang, J. Ma and X. Lu, *ACS Appl. Mater. Interfaces*, 2011, **3**, 782–788. Copyright 2011 American Chemical Society.

It was found that the PANI-SWCNT had a lower oxidation potential of 0.10 V compared to PANI/PSS which has an oxidation potential of 0.22 V as well as a larger charge storage capacity. This was attributed to the conductive function of the SWCNTs. The SWCNT also enhances the electrochromic properties of PANI. The electrochromic data for neat PANI and the various PANI-SWCNT hybrids is shown in Table 6. Upon oxidation, normal PANI experiences a blue shift in *λ*_max_ from 778 nm at −2 V to 680 nm at 2 V with an OC of 34% at 680 nm and a CE of 105 cm^2^ C^−1^ while PANI with 0.8% SWCNT incorporation results in a larger blue shift from 768 nm at −2 V to 630 nm at 2 V, a higher OC of 47% at 630 nm, and a larger CE of 168 cm^2^ C^−1^. Thus, 0.8% SWCNT incorporation resulted in a 50 nm blue shift of oxidized *λ*_max_ and an enhancement in OC and CE for PANI. This was ascribed to the increased doping and improved electron transport provided by the SWCNTs. The colours of the device switched from greenish yellow at −2 V to sky blue at 2 V. The enhancements provided by the SWCNTs also led to improved switching kinetics. PANI-SWCNT had steeper and flatter switching curves indicating faster colouration and bleaching times than unmodified PANI (Fig. 18). Overall, this work demonstrates how simply covalently linking PANI to a substrate like SWCNT without any further modification to PANI can lead to significant improvements in electrochromic behaviour.

**Table 6:** Electrochromic data for PANI and PANI-SWCNT hybrids^43^

	PANI	PANI-SWCNT_0.2%_	PANI-SWCNT_0.4%_	PANI-SWCNT_0.8%_
*λ*_max_ (nm) −2 V	778	775	772	768
*λ*_max_ (nm) 2 V	680	660	655	630
OC (%)	34	36	43	47
CE (cm^2^ C^−1^)	105	114	159	168

**Fig. 19:**
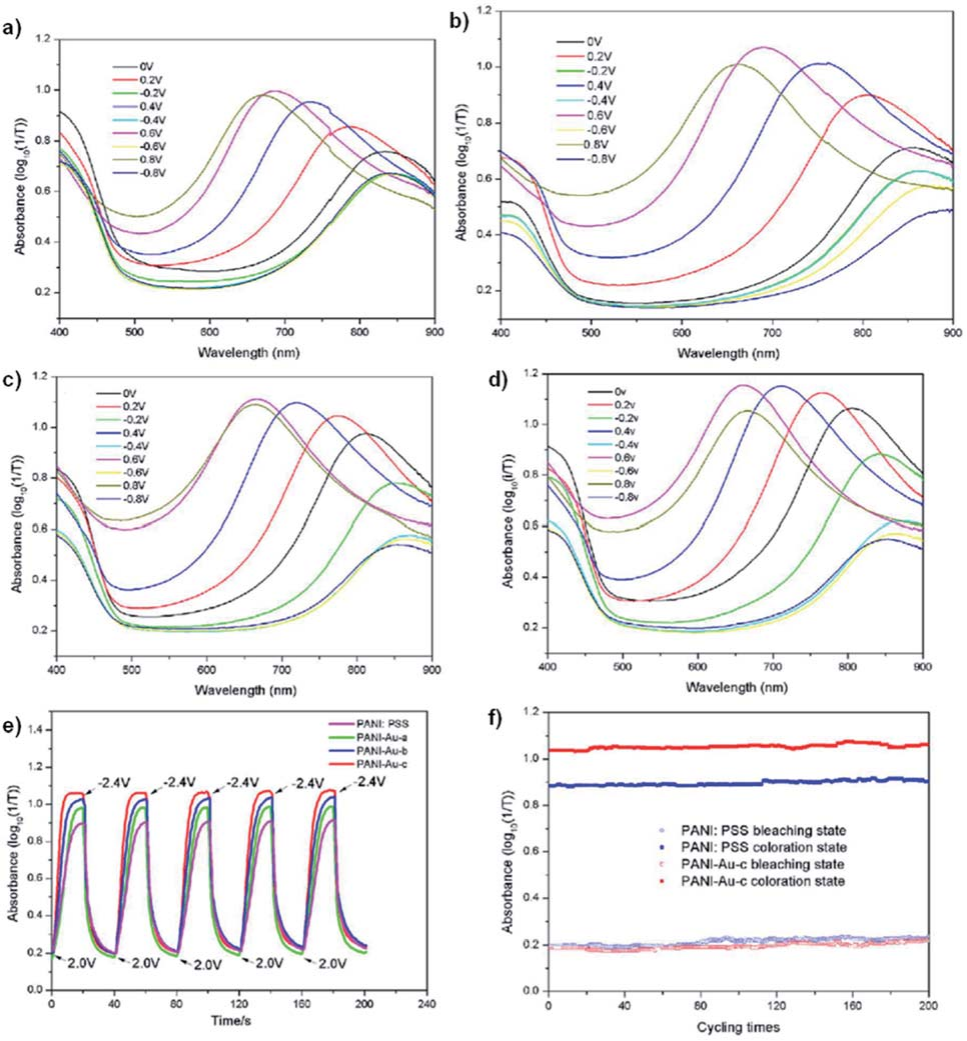
UV-vis absorbance of PANI/PSS (a), PANI-Au-a (b), PANI-Au-b (c) and PANI-Au-c (d) under different potentials. (e) Optical absorbance at *λ*_max_ for the devices with PANI/PSS and PANI-Au hybrids as the electrochromic layers and (f) the cycling stability of PANI/PSS and PANI-Au-c based devices under the step potential oscillating between 2.0 V and −2.4 V with a 40 s interval. The PANI-Au hybrids possess gold to aniline molar ratios of 1 : 200, 1 : 100, and 1 : 50 for PANI-Au-a, PANI-Au-b, and PANI-Au-c, respectively. Reprinted from *Sol. En. Mat. Sol. C*, vol. **177**, S. Xiong, J. Lan, S. Yin, Y. Wang, Z. Kong, M. Gong, B. Wu, J. Chu, X. Wang, R. Zhang, and Y. Li, Enhancing the electrochromic properties of polyaniline *via* coordinate bond tethering the polyaniline with gold colloids, pages 134–141, Copyright 2018, with permission from Elsevier.

Carbon nanotubes have not been the only conductive material used to enhance PANI's electrochromic properties. Xiong *et al.* has also tethered PANI to gold nanoparticles to create an organic-inorganic hybrid electrochromic material, PANI-Au.^44^ These were prepared by reducing HAuCl_4_ in water to form gold colloids which were then functionalized with *p*-aminothiophenol in ethanol. Polymerization with aniline monomer was then performed in water with PSS and APS. Gold nanoparticles are known to be strongly coloured ranging from red to bluish purple depending on their size.^80^ The gold nanoparticles synthesized were ∼30 nm which corresponded to a red purple colour with a *λ*_max_ of 524 nm. The *p*-aminothiophenol was used to provide a bridging agent between the gold colloids and the polymer, much like the amide of the previous study, as sulfur is known to strongly coordinate to gold. And as in the previous study, the gold was found to enhance the electronic properties of PANI shifting oxidation peaks to lower potentials and reduction peaks to higher potentials depending on amount of gold compared to PANI/PSS. Thus, the PANI-Au nanohybrids are more easily oxidized and reduced than PANI/PSS and can change colour at lower potentials. Oxidation resulted in colour change from yellow-green to sky blue for PANI/PSS and PANI-Au with PANI/PSS achieving its maximum doping state at 0.8 V with an optical contrast of 76% while PANI-Au achieved its maximum doping state at 0.6 V with an optical contrast of 97%. The absorbance *λ*_max_ of the oxidized state also blue-shifted from 690 nm for PANI/PSS to 660 nm for PANI-Au. The switching times were also enhanced with colouration and bleaching times decreasing from 13.4 s and 8.9 s for PANI/PSS, respectively, to 8.3 s and 7.6 s for PANI-Au, respectively. Thus, as in the previous study, affixing PANI to a highly conductive material like gold nanoparticles without any further modification to the polymer is sufficient to achieve large improvements in electrochromic properties (Fig. 19).

### 4.3. Perylene bisimides

As discussed above, perylene bisimides (PBIs), also referred to in literature as perylene diimides (PDIs) and perylene tetracarboxylic diimides (PTCDIs), are commonly used n-type semiconducting materials in a wide variety of organic electronic devices, including ECDs. Whereas NDIs are colourless in the neutral state and are Type I electrochromes, PBIs are vibrantly coloured in both neutral and reduced states making them Type II electrochromes. Owing to their unique optoelectronic properties and ease of functionalization, many research groups have developed a wide variety of water soluble PBIs for use in organic electronics.

**Fig. 20:**
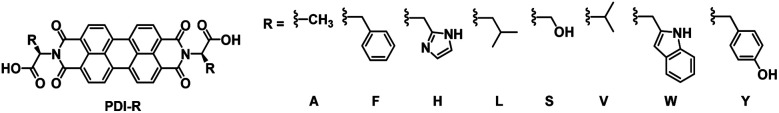
Water soluble PBIs prepared by our group.^81^

**Fig. 21:**
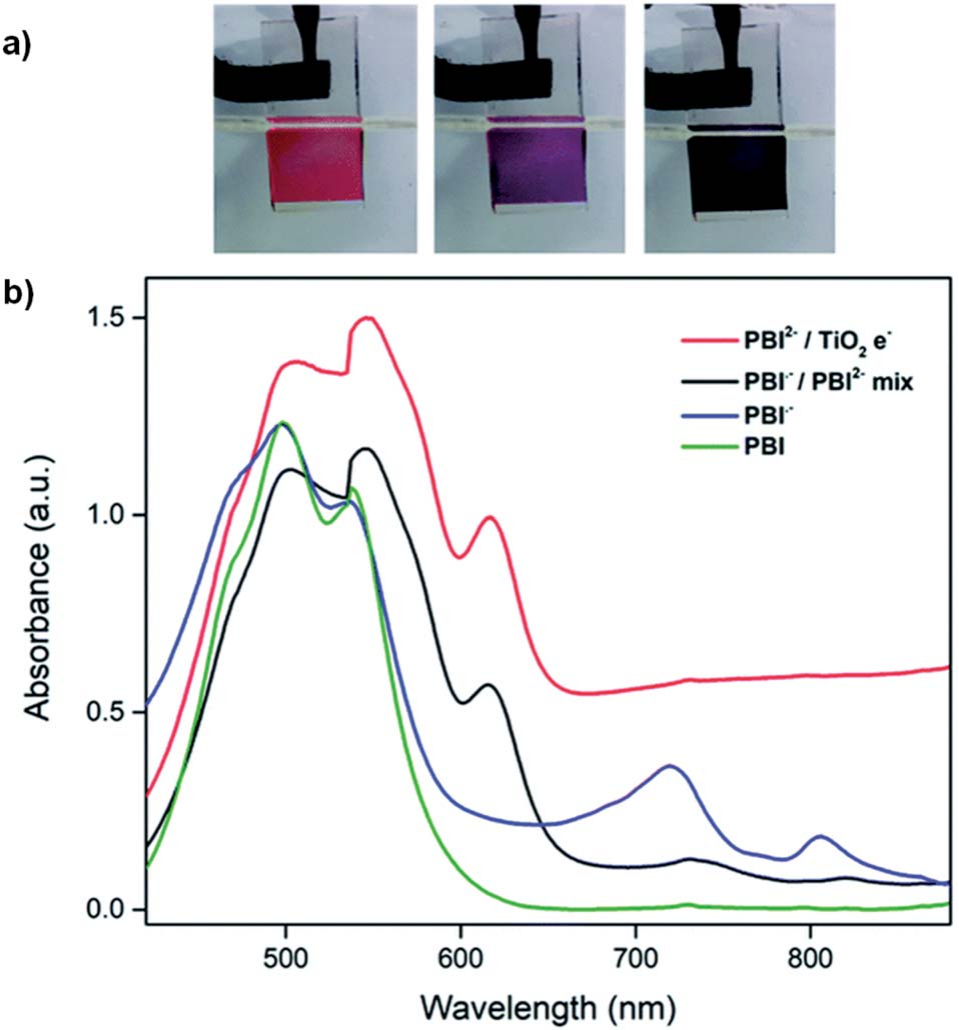
(a) Films of TiO_2_/PBI-A at 0 V (red), −1.0 V (purple) and −1.5 V (navy blue). (b) Absorption profiles for PBI-A neutral, radical anion, and dianion forms and with reduced with TiO_2_. Reproduced from ref. 45 with permission from the Centre National de la Recherche Scientifique (CNRS) and The Royal Society of Chemistry.

Following our work with amino acid-functionalized electrochromic NDIs, we have also prepared several amino acid-functionalized water soluble PBIs (Fig. 20).^81^ One of these, PBI-A bearing an alanine side group, was coordinated to TiO_2_ nanoparticles to enhance the PBI's electrochromic properties and make it suitable for device application.^45^ Previous studies with PBI-A have shown that it forms aggregates in aqueous solution to form well defined helical bundles depending on concentration as well as solution pH.^82^ When appended onto TiO_2_ the PBI-A forms similar aggregates as indicated by the UV/Vis absorption spectra. Soaking TiO_2_-on-FTO films in 10^−4^ M PBI-A aqueous solutions at pH 6.1 resulted in pink films of TiO_2_/PBI-A with *λ*_max_ at 500 nm and 550 nm. Analysis by ATR-FTIR indicated coordination occurred *via* the PBI-A carboxylate groups to TiO_2_. The electrochromic properties of the TiO_2_/PBI-A films were measured and it was found that reduction at −1.0 V led to the film turning purple from the formation of PBI-A radical anion which absorbs at 715 nm. Further reduction at −1.5 V turned the films navy blue, the colour of reduced TiO_2_. The absorption profiles and colour changes for TiO_2_/PBI-A films are shown in Fig. 21. The OC at 715 nm was found to be 32% with a CE of 37 cm^2^ C^−1^. In contrast, PBI polymerized directly onto FTO was found to have an OC of 29% and CE of 295 cm^2^ C^−1^.^83^ Electrochromic switching was also performed for our TiO_2_/PBI-A on FTO films with monitoring at 715 nm. After 500 switching cycles there was a drop in conductivity and the CV response changed from reversible to irreversible indicating degradation of the films. The lower CE and low stability were attributed to the degradation of the underlying FTO layer in the films. TiO_2_/PBI-A by itself was found to be very stable as indicated by the UV/Vis absorption spectrum of TiO_2_/PBI-A being unchanged after cycling without any indication of degradation. This shows that TiO_2_/PBI-A has excellent potential for application in ECDs, assuming the stability of FTO can be improved or a substitute found.

**Fig. 22:**
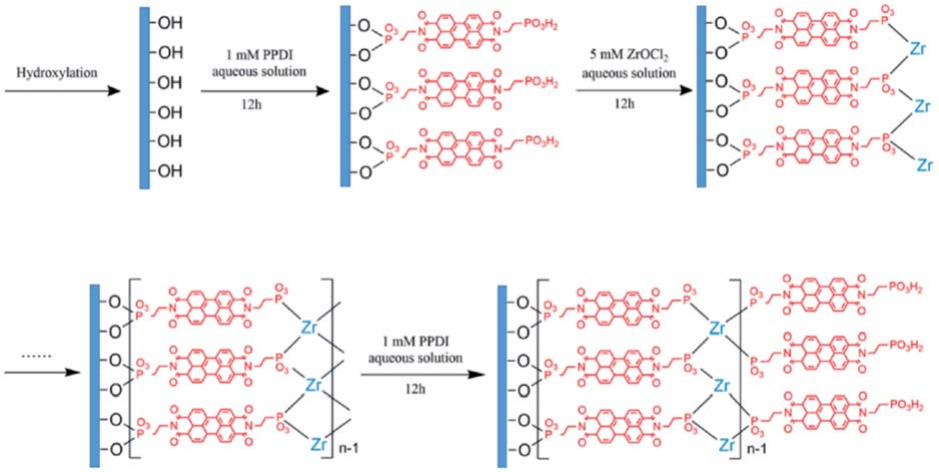
Self-assembly of PPBI films on hydroxylated ITO-coated glass. Reprinted from Chem. Eng. J., vol. 386, X. Lv, L. Zha, L. Qian, X. Xu, Q. Bi, Z. Xu, D. S. Wright and C. Zhang, controllable fabrication of perylene bisimide self-assembled film and patterned all-solid-state electrochromic device, Page 123939, Copyright 2020, with permission from Elsevier.

A recent example of a water soluble PBI comes from Lv *et al.* who reported on PBI functionalized with phosphate groups at the imides.^46^ The PPBI could self-assemble into films with zirconium ions and this was used to fabricate ECDs using a layer-by-layer fabrication method. Hydroxylated ITO-coated glass was submerged in a 1 mM aqueous solution of PPBI for 12 h to form a layer of PPBI. This was then submerged in a 5 mM aqueous solution of ZrOCl_2_ to form a layer of Zr ions on top of the PPBI. The process was repeated to build films with 4, 6, 8, 10, and 12 layers (Fig. 22).

**Fig. 23:**
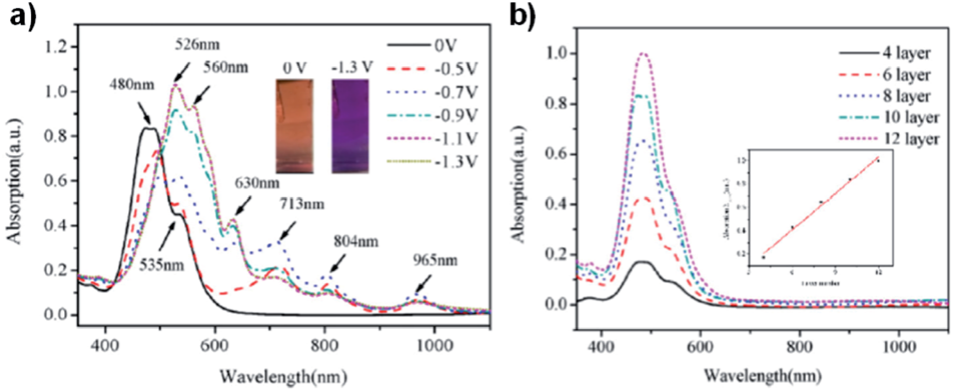
(a) Absorption spectra of PPDI self-assembled films with 10 layers at various applied potentials in 0.2 M [BMIM]OTf/PC solution. (b) Absorption spectra of PPBI self-assembled films with different numbers of layers in 0.2 M [BMIM]OTf/PC solution. Reprinted from *Chem. Eng. J.*, vol. **386**, X. Lv, L. Zha, L. Qian, X. Xu, Q. Bi, Z. Xu, D. S. Wright and C. Zhang, Controllable fabrication of perylene bisimide self-assembled film and patterned all-solid-state electrochromic device, page 123939, Copyright 2020, with permission from Elsevier.

The films were orange-red with absorption bands at 480 nm and 535 nm. Upon reduction at −0.5 V new bands appeared at 713, 814, and 965 nm. These bands were associated with the PBI radical anion. Further reduction at −1.3 V led to new bands at 526, 560, and 630 nm for the PBI dianion and the films became deep purple (Fig. 23). The number of layers influenced the OCs, switching times, and CEs (Table 7). The film with 10 layers was found to have had reasonable properties all round with a 32% OC, 0.75 s colouration time, 2.31 s bleaching time, and 80.51 cm^2^ C^−1^ CE. These parameters were determined by monitoring at 630 nm and switching times and CEs were defined at 95% of the transmittance change. The stability of this film was also determined, and it was found to show no change in OC after 6000 cycles of switching between 0 V and −1.3 V with a 5 s residence time. The PPBI/Zr four layers film was incorporated in an all-solid-state patterned ECD using the gel electrolyte PMMA/[BMIM]OTf/PC (PMMA = poly(methylmethacrylate), [BMIM]OTf = 1-butyl-3-methylimidazolium trifluoromethane-sulfonate, PC = propylene carbonate). This device switched from orange-red to deep purple with application of −3.5 V and switched back at 1.0 V with a higher OC of 46.8% due to improved film stability in the solvent and oxygen free environment. Patterned devices were fabricated by using a mask when preparing the conductive substrate prior to film formation. The high OC and ability to form patterned devices means this PPBI has great potential in applications such as smart windows, sunglasses, and electronic tags.

**Table 7:** Electrochromic data for self-assembled PPBI/Zr films measured at 630 nm^46^

Layers	OC (%)	CE (cm^2^ C^−1^)	Response times (s)	
			Colouration	Bleaching
4	12	63.53	0.34	1.57
6	20	76.72	0.52	1.68
8	25	77.65	0.60	1.94
10	32	80.51	0.75	2.31
12	41	100.42	1.01	2.54

### 4.4. Electro-acid/bases

**Fig. 24:**
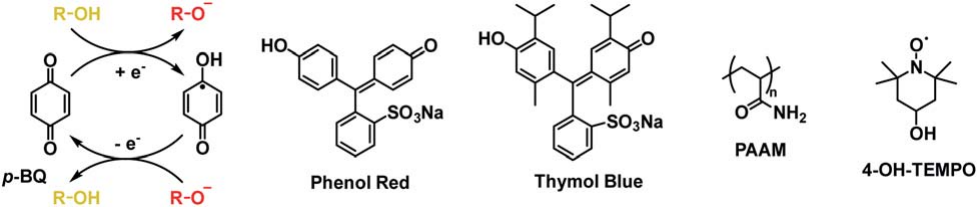
The mechanism by which electro-acid/base occurs and the materials used to fabricate the electrochromic hydrogel.^47^

An alternative to PANI and PBI in Type II electrochromic devices can be found in a recent report from Yang *et al.* who developed a wearable ECD based on an intrinsically stretchable hydrogel with common pH indicators and a reversible “electro-acid/base.“^47^ An electro-acid/base is a compound that undergoes a proton transfer upon oxidation or reduction.^84–86^ In the presence of an indicator such as phenol red or thymol blue, this results in a colour change. In this study the researchers used *p*-benzoquinone (*p*-BQ), a water soluble compound that accepts a proton when reduced, thereby increasing the pH of its solution. To fabricate the ECD a hydrogel was first prepared from polyacrylamine (PAAM). This hydrogel was then swelled with an aqueous solution containing *p*-BQ, the desired indicator, KCl as an electrolyte, and 4-OH-TEMPO as an ion storage material to form the electrochromic hydrogel. Phenol red sodium salt, which is yellow at low pH and red at high pH, and thymol blue sodium salt, yellow at low pH and blue at high pH, were chosen as the indicators for their excellent water solubilities and well defined colour changes. Moreover, the reduction potentials of phenol red and thymol blue are below −0.60 V while *p*-BQ reduces at −0.12 V, meaning the indicators would not be reduced during the reduction of *p*-BQ. The mechanism, shown in Fig. 24, of the electro-acid/base occurs *via* reduction of *p*-BQ to the radical anion *p*-BQ˙^−^ which abstracts a proton from the indicator thereby changing the colour. This process is reversible by oxidation of *p*-BQH˙ which gives the proton back to the indicator reversing the colour change.

**Fig. 25:**
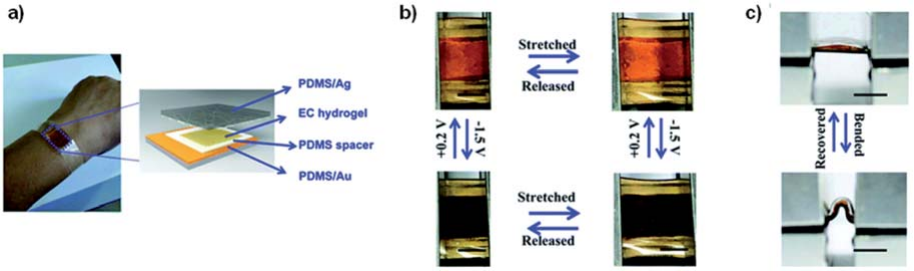
(a) Wearable ECD with schematic diagram. (b) Display of the phenol red hydrogel ECD's deformable performance. (c) Display of ECD being bent. Reproduced from ref. 47 with permission from The Royal Society of Chemistry.

It was found that upon application of −0.5 V the phenol red hydrogel changed from yellow (*λ*_max_ of 438 nm) to purple (*λ*_max_ 559 nm) and the thymol blue hydrogel change from yellow (*λ*_max_ 438 nm) to blue (*λ*_max_ 595 nm), the same colour changes induced by addition of NaOH to solutions containing just the indicators. These hydrogels were sandwiched between ITO-coated glass with a polydimethylsiloxane spacer to fabricate ECDs. The phenol red device had a CE of 291.35 cm^2^ C^−1^ with colouration and bleaching times of 10.89 s and 11.46 s, respectively, while the thymol blue device had a CE of 152.18 cm^2^ C^−1^ with colouration and bleaching times of 8.44 s and 9.02 s, respectively. Switching times were defined at 90% transmittance change. Both devices maintained their performance over 200 cycles with minimal loss in OC. Wearable flexible devices were also fabricated with the hydrogels using Au nanosheets as stretchable anodes and Ag nanowires as flexible cathodes (Fig. 25). It was found that these devices could maintain their performance when subjected to a 20% strain for over 1000 stretching cycles. With 50% strain there was an irreversible loss in conductivity attributed to the Au nanosheets breaking. Overall, this study demonstrated an effect method by which to fabricate flexible ECDs. The colours can be easily modified by changing the type of indicator or electrochrome used in the hydrogel which has important implications for application in wearable devices and smart windows.

## 5. Conclusions

Using organic materials in electrochromic devices affords a number of advantages over more traditional transition metal oxides such as WO_*x*_. Organic materials are flexible, can be easily modified to tune their optoelectronic properties providing a wider range of colours, and can be derived from renewable feedstocks. However, unlike transition metal oxides, organic materials are not usually water soluble. The development of more water soluble organic electronic materials is of paramount importance for environmental and economic sustainability. This review has highlighted water soluble electrochromic organic materials that display an array of colours and promising device parameters including reversibility and cyclability in the hopes of promoting these materials and inspiring future researchers to develop more water soluble materials for a wide range of organic electronic applications. Organics materials often suffer from the perception that they are less stable, have low cyclabilities, and require toxic synthetic methods to produce compared to inorganics. The examples provided in this review have shown that this perception is simply not true.

Both polymeric and molecular water soluble electrochromes have been discussed and these can be further distinguished into two classes: electrochromes that switch between colourless and coloured states (Type I) such as PEDOT, PProDOT, viologens, and NDIs; and electrochromes that switch between differently coloured states (Type II) such as polythiophenes, PANI, and PBIs. For both types of electrochromes, water solubility is very important for their future application by reducing the costs and hazards in manufacturing. As shown by the examples above, there are already several promising applications including in smart windows, wearable devices, and multicoloured displays. Type I electrochromic materials and their devices are of primary interest in the development of smart windows where a fully transparent and colourless state is necessary while Type II are of greater interest in display technologies such as smart screens. It is Type I electrochromic materials and the development of smart window technology that holds the most promise for future research. Smart windows can be used in the transportation sector to reduce glare in cars or airplanes as well as in the construction sector to make more energy efficient buildings. Type II electrochromic materials, while still worth pursuing for their application in display systems, face considerable competition from OLED technology in that area. As shown in Table 1, the majority of Type I electrochromes change between colourless and blue states. Thus, future developments should focus on expanding the range of colours that materials can produce. In particular, materials that can change between a colourless, fully transparent state and a black, fully opaque state is of particular interest in smart window technology for privacy purposes.

Future developments should also focus on expanding the types of electrochromes that can be made water soluble. As shown in this review, many water soluble electrochromic materials are based on viologens and, because of their inherent toxicity, are limited in their widescale application. Developing more water soluble materials with less harmful properties would alleviate that concern. Our group, among many others, has shown the ease with which insoluble NDIs or PBIs can be made water soluble through simple functionalization. NDIs and PBIs have the added benefit of low toxicity and are readily available from renewable feedstocks. More efforts should be devoted to making other organic materials water soluble in order to reduce the concerns with using environmentally harmful synthetic and processing conditions. Future efforts should also address issues that arise with processing from aqueous media. Casting optical-level thin films from aqueous formulations is currently very difficult which limits the types of devices that can be made with water soluble materials. More research with water soluble materials will undoubtedly lead to improvements in processing that overcome these issues. Overall, the future for water soluble organic electrochromic technology is very promising and will only become more relevant as the need for environmentally friendly and cost-effective methods of material synthesis and processing increases.

## Conflicts of interest

There are no conflicts of interest to declare.

## Supplementary Material
